# Automatic morpheme identification across development: Magnetoencephalography (MEG) evidence from fast periodic visual stimulation

**DOI:** 10.3389/fpsyg.2022.932952

**Published:** 2022-09-07

**Authors:** Valentina N. Pescuma, Maria Ktori, Elisabeth Beyersmann, Paul F. Sowman, Anne Castles, Davide Crepaldi

**Affiliations:** ^1^Cognitive Neuroscience, International School for Advanced Studies (SISSA), Trieste, Italy; ^2^School of Psychological Sciences, Macquarie University, Sydney, NSW, Australia; ^3^Macquarie University Centre for Reading, Macquarie University, Sydney, NSW, Australia

**Keywords:** magnetoencephalography, fast periodic visual stimulation, morphological processing, automatic morpheme identification, reading development, English

## Abstract

The present study combined magnetoencephalography (MEG) recordings with fast periodic visual stimulation (FPVS) to investigate automatic neural responses to morphemes in developing and skilled readers. Native English-speaking children (*N* = 17, grade 5–6) and adults (*N* = 28) were presented with rapid streams of base stimuli (6 Hz) interleaved periodically with oddballs (i.e., every fifth item, oddball stimulation frequency: 1.2 Hz). In a manipulation-check condition, tapping into word recognition, oddballs featured familiar words (e.g., *roll*) embedded in a stream of consonant strings (e.g., *ktlq*). In the experimental conditions, the contrast between oddball and base stimuli was manipulated in order to probe selective stem and suffix identification in morphologically structured pseudowords (e.g., stem + suffix pseudowords such as *softity* embedded in nonstem + suffix pseudowords such as *trumess*). Neural responses at the oddball frequency and harmonics were analyzed at the sensor level using non-parametric cluster-based permutation tests. As expected, results in the manipulation-check condition revealed a word-selective response reflected by a predominantly left-lateralized cluster that emerged over temporal, parietal, and occipital sensors in both children and adults. However, across the experimental conditions, results yielded a differential pattern of oddball responses in developing and skilled readers. Children displayed a significant response that emerged in a mostly central occipital cluster for the condition tracking stem identification in the presence of suffixes (e.g., *softity* vs. *trumess*). In contrast, adult participants showed a significant response that emerged in a cluster located in central and left occipital sensors for the condition tracking suffix identification in the presence of stems (e.g., *softity* vs. *stopust*). The present results suggest that while the morpheme identification system in Grade 5–6 children is not yet adult-like, it is sufficiently mature to automatically analyze the morphemic structure of novel letter strings. These findings are discussed in the context of theoretical accounts of morphological processing across reading development.

## Introduction

Morphemes are the smallest linguistic units that bear meaning. For instance, a complex word like *artist* contains a stem, *art-*, and a suffix, *-ist*. Many languages are morphologically rich, meaning that their lexicon includes a great deal of complex words, by derivation, inflection, or compounding; it is estimated that 85% of the English lexicon is made up of complex words (Algeo and Algeo, 1993; Grainger and Ziegler, 2011).

Considering the important role that efficient morphological processing plays in skilled reading (Rastle, 2019), it is unsurprising that many studies in the psycholinguistic domain have focused on the sensitivity to morphological structure during visual word processing (for a review, see Amenta and Crepaldi, 2012). Several theories have been proposed over the years to account for the visual identification, comprehension, and reading aloud of complex words. Some of these theories dispose entirely of explicit morphological representations, and trace back the emergence of morphological effects to the appreciation of statistical regularities in mappings between form, meaning, and phonology (e.g., Seidenberg, 1987; Baayen et al., 2011; for a review, see Stevens and Plaut, 2022). Other *localist* models affirm the existence of morphological representations, either through different, serially arranged stages of processing (e.g., Crepaldi et al., 2010; Taft and Nguyen-Hoan, 2010; Taft, 2015), or along parallel routes (e.g., Grainger and Ziegler, 2011). More recently, Grainger and Beyersmann (2017; see also Beyersmann and Grainger, 2022) proposed that the analysis of the internal structure of words is initiated by the identification of stems as embedded, edge-aligned words. Although the cognitive architecture implied by this model is not substantially different from its predecessors, Grainger and Beyersmann’s theoretical account is new in the proposal of a lexical trigger (i.e., the identification of word stems) for morphological analysis, as well as in the different mechanisms it assigns to the identification of stems and affixes. Notably, localist models of morphology build in different ways on the distinction between a level of morphological processing that is mostly based on form and one in which meaning plays a more substantial role.

Indeed, there is wide evidence that skilled reading is characterized by a rapid and automatic process of morphological analysis that operates on any printed word that merely has the orthographic appearance of being complex (Rastle et al., 2004). The main support for this so-called morpho-orthographic processing comes from masked priming studies. This research has found that adult readers routinely show facilitation not only for pairs of words with a semantically-transparent morphological relationship (e.g., *reader* primes the recognition of READ) but also for pairs with a pseudo-morphological relationship (e.g., *corner* primes CORN, relative to a purely orthographic baseline with no apparent morphological structure, e.g., *brothel-BROTH;* e.g., Longtin et al., 2003; Rastle et al., 2004; Beyersmann et al., 2016; see Rastle and Davis, 2003, for a review).

Interestingly, these behavioral findings are bolstered by neurophysiological studies (see Leminen et al., 2019, for an extensive review) examining the time course and neural bases of morphological processing. For example, Whiting et al. (2015), conducted a masked priming MEG study to investigate differences in the processing of simple (*walk*), complex (*farmer*), and pseudocomplex (*corner*) words. For both complex and pseudocomplex items, a similar morphological effect emerged around 330–340 ms in the left middle temporal gyrus (MTG), diverging from noncomplex stimuli. This pattern of findings suggests that both complex and pseudocomplex items undergo a “blind” decomposition process, reflecting morpho-orthographic processing (see also Lavric et al., 2011; Beyersmann and Grainger, 2022, for similar EEG evidence). This is further corroborated by fMRI evidence, such as the masked priming study by Gold and Rastle (2007), in which a similar pattern of reduced activation was observed in the left posterior middle occipital gyrus for pseudomorphologically related pairs (*archer-ARCH*) and for orthographically related ones (*pulpit-PULP*), and reduced activity of the posterior face fusiform gyrus was observed specifically for pseudomorphologically related pairs.

### Morphological processing across reading development

Children as young as 7 years show evidence for explicit morphological knowledge. They can successfully manipulate and reflect on the morphological structure of words and novel letter strings, as measured by various types of morphological awareness tasks (e.g., Kirby et al., 2012). Furthermore, there is substantial evidence that young readers’ morphological knowledge implicitly influences their online performance on word reading and recognition tasks (Rastle, 2019). For example, Carlisle and Stone (2005) showed that children in the initial (Grades 2 and 3) and later (Grades 5 and 6) years of primary school read aloud real morphologically complex words (e.g., *hilly*) more accurately than pseudo-morphological words, matched on number of syllables, spelling, and frequency (e.g., *silly*). Likewise, Burani et al. (2002) showed that Italian children between Grades 3 and 5 read aloud morphologically structured pseudowords (e.g., *donnista*, “womanist,” composed of the root *donn-*, “woman” plus the suffix *-ista*, “-ist”) more rapidly and accurately than pseudowords without a morphological structure (e.g., *dennosto*, composed of the non-root *denn-* plus the non-suffix *-osto*). When participating in a lexical decision task, the same children also showed greater difficulty in rejecting morphologically structured pseudowords; a morpheme interference effect that has since been replicated with children of similar school grades in French (Casalis et al., 2015) and in English (Dawson et al., 2018).

But at what stage of reading development does the ability to recognize morphemes rapidly and automatically emerge? To address this question, a series of masked priming studies sought direct evidence for morpho-orthographic processing in developing readers. The evidence they provided, however, is rather mixed. For example, using masked priming, Beyersmann et al. (2012) found that although English third and fifth graders showed facilitation for morphologically related pairs (e.g., *golden-GOLD*), there was no evidence for priming between pairs of words sharing pseudo-morphological (e.g., *mother-MOTH*) or purely orthographic (e.g., *spinach-SPIN*) overlap. But a different pattern of results emerged in a study by Quémart et al. (2011) with French-speaking children. In this experiment, third, fifth, and seventh graders yielded similar priming effects for opaque (*baguette-BAGUE*) and transparent pairs (*tablette-TABLE*), but no priming for orthographic (*abricot-ABRI*) or semantic (*tulipe-FLEUR*) pairs. Yet Schiff et al. (2012) found a different set of results in Hebrew, with fourth and seventh graders showing equally strong priming for prime and target pairs that were morphologically and semantically related, and seventh graders showing additionally a weak priming effect for pairs that were morphologically related and semantically unrelated—a pattern similar to that observed with adult readers of Hebrew (Bentin and Feldman, 1990; Frost et al., 1997).

More recently, Dawson et al. (2018) carried out a more fine-grained investigation into the emergence of adult-like morphological processing in English by including adolescent readers. Using unprimed lexical decision, they showed that although all groups of English-speaking participants rejected pseudo-morphemic pseudowords (e.g., *earist*) less accurately than control pseudowords (e.g., *earilt*), this difference was greater in adults and older adolescents (16–17 years) than in younger adolescents (12–13 years) and children (7–9 years). Furthermore, only adults and older adolescents exhibited a morpheme interference effect in their response times. Together these findings suggest that the way morphological representations are used in visual word recognition continues to undergo important changes during adolescence.

In summary, there is substantial evidence that within only a few years of reading instruction children demonstrate sensitivity to morphological structure during visual word processing. Yet, it remains unclear at what stage in reading development morphological processing is automatized. The available developmental data provide a mixture of results, with some recent evidence indicating that the morpheme recognition processes continue to develop even during adolescence. Admittedly, conclusions are further hindered by the different languages in which this research has been conducted. Indeed, the developmental trajectory of morphological processing appears to differ across languages, a claim that has found support in several recent cross-linguistic investigations (e.g., Beyersmann et al., 2020, 2021b; Mousikou et al., 2020). Another possibility is that the lack of clear evidence is due, at least in part, to issues related to commonly used behavioral paradigms, often requiring children to sit through long sessions and perform a somewhat unnatural task (e.g., primed or unprimed lexical decision), usually yielding quite small effects. One could, of course, take recourse to neurophysiological evidence to resolve this type of inconsistencies. However, to our knowledge, such developmental evidence is nonexistent. To overcome these limitations, the present study seeks to investigate automatic morpheme identification in developing readers by capitalizing on a relatively novel, behavior-free technique that combines Fast Periodic Visual Stimulation (FPVS) with electrophysiological recordings (Rossion, 2014).

### Fast periodic visual stimulation (FPVS) and visual word recognition

The FPVS approach is based on the principle of neural entrainment (see Norcia et al., 2015, for a review), and when applied in the context of an oddball paradigm, it relies on frequency tagging to effectively capture visual discrimination processes at the level of the brain. This usually involves presenting sequences of base stimuli at a fast periodic rate (i.e., base stimulation frequency *F*) with oddball stimuli periodically inserted at fixed intervals within the stream (every n^th^ item), thus resulting in a slower presentation rate (i.e., oddball stimulation frequency *F/n*). A peak in the neural signal at the oddball stimulation frequency (and its harmonics) indexes the brain’s ability to successfully discriminate between oddball and base stimuli. Critically, oddball responses are selective to the dimension that differentiates oddballs from base stimuli.

To date, the FPVS-oddball paradigm has been most commonly used to investigate face processing and recognition (e.g., Dzhelyova and Rossion, 2014; Rossion, 2014; Rossion et al., 2015; Retter and Rossion, 2016; Quek et al., 2018). However, thanks to its versatility, it has gained popularity in many other areas of cognitive processing, including visual word recognition. For example, Lochy et al. (2015) combined FPVS and EEG recordings to probe selective neural representations of words (relative to pseudowords) in skilled adult readers. Even more relevant to the present study, however, the FPVS approach enjoys several advantages that make it ideal for special populations like children. Specifically, the approach is highly sensitive such that only a few minutes of stimulation are sufficient to elicit robust responses with a high signal-to-noise ratio (SNR). This diminishes the need for a large number of experimental trials, especially when small effects are considered. Furthermore, the neural responses elicited by FPVS are clearly and objectively identifiable in the pre-defined base and stimulation frequencies, thus eliminating the subjectivity that can, at times, accompany the detection of event-related components. Finally, the approach does not require participants to actively engage with the experimental stimuli. As such, neural discrimination responses are obtained implicitly and automatically, and are devoid of potential contamination from task-induced cognitive and decisional processes. In this respect, Lochy et al. (2016) already provide us with proof of concept by successfully combining FPVS with EEG recordings to elicit selective neural responses to letter strings in young preschoolers. Here, we pair this technique with magnetoencephalography (MEG) to investigate for the first time an even more fine-grained level of visual word processing, namely the identification of morphemes, across reading development.

### The present study

**Figure 1:**
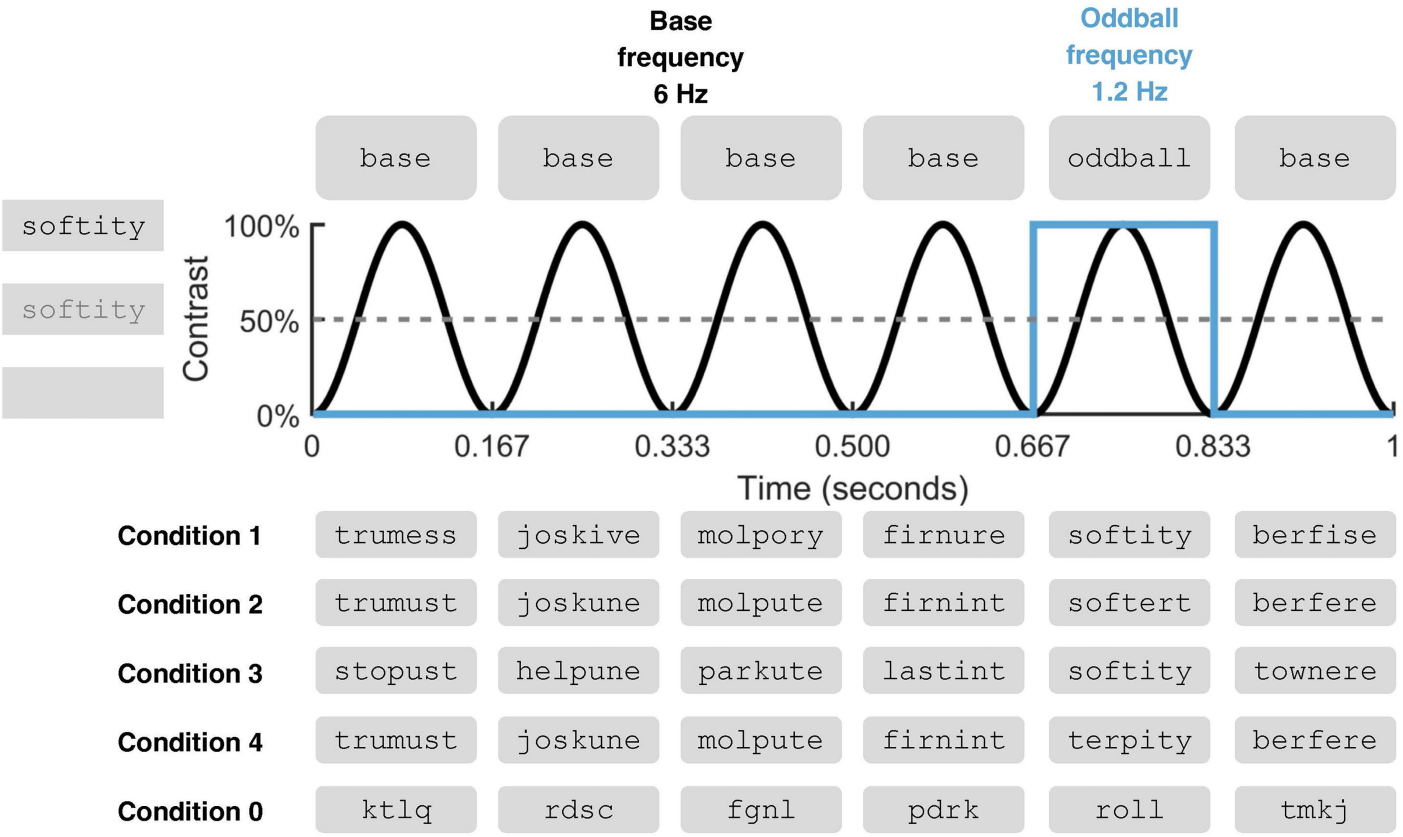
Fast Periodic Visual Stimulation (FPVS) in the context of an oddball paradigm (figure adapted from De Rosa et al., 2022). The schematic illustration pertains to one second of stimulation. For a gradual and smooth transition between them, stimuli were presented *via* sinusoidal contrast modulation at 6 Hz during 60 s, with each cycle reaching full contrast after 83.5 ms and lasting a total of 167 ms. Oddball stimuli appeared every fifth item (6/5 = 1.2 Hz). Examples are given for the different types of sequences used in the experimental conditions. To examine stem identification, Condition 1 featured stem + suffix oddballs embedded in a stream of nonstem + suffix pseudowords, and Condition 2 (adult sample only) featured stem + nonsuffix oddballs embedded in a stream of nonstem + nonsuffix pseudowords. To examine suffix identification, Condition 3 featured stem + suffix oddballs embedded in a stream of stem + nonsuffix pseudowords and Condition 4 (adult sample only) featured nonstem + suffix oddballs embedded in a stream of nonstem + nonsuffix pseudowords. A manipulation-check condition (Condition 0) was administered to all participants and examined a selective neural response to words that appeared as oddballs within a sequence of unpronounceable letter strings. During stimulation, participants engaged in the independent task of monitoring and responding to changes in the color of a fixation cross at the center of the screen.

In the present FPVS-MEG study, we presented a group of native English-speaking children (Grades 5 and 6) and a group of native English-speaking adults with rapid sequences of carefully constructed pseudowords in order to examine automatic neural responses to morphemes. By definition, pseudowords are not represented in the lexicon. As such, they constitute ideal linguistic stimuli to explore morpho-orthographic analysis that is considered to operate rapidly and automatically on any printed letter string prior to lexical access (Taft and Forster, 1975). The experimental stimuli consisted of four types of pseudowords. We manipulated the contrast between oddball and base stimuli in order to probe selective stem and suffix identification (see Figure 1). Specifically, to investigate stem identification, two of the experimental conditions featured oddball pseudowords that were either composed of a real English stem and a real English suffix (e.g., *softity*; Condition 1) or of a stem only (e.g., *softert*; Condition 2). To investigate suffix identification, two additional experimental conditions featured oddball pseudowords that were either composed of a real English stem and a real English suffix (e.g., *softity*; Condition 3) or of a suffix only (e.g., *terpity*; Condition 4). In order to elicit a contrast, oddballs were embedded in streams of base stimuli which did not contain the critical morpheme. Namely, in Condition 1, the stem + suffix oddballs (*softity*) were embedded in streams of nonstem + suffix base stimuli (*trumess*), so that the contrast between the two would track stem identification, in the presence of a suffix. In Condition 2, the stem + nonsuffix oddballs (*softert*) were embedded in streams of nonstem + nonsuffix base stimuli (*trumust*), so that the contrast would still track stem identification, this time in the absence of a suffix. Similarly, Conditions 3 and 4 featured, respectively, stem + suffix oddballs embedded in stem + nonsuffix base stimuli (*softity* vs. *stopust*), and nonstem + suffix oddballs embedded in nonstem + nonsuffix base stimuli (*terpity* vs. *trumust*), thus tracking suffix identification, either in an exhaustively decomposable morphological context or not.

In the current design, a robust oddball response across Conditions 1 and 2, and/or across Conditions 3 and 4, would be suggestive of sensitivity to the individual morphemes regardless of whether the oddball can be exhaustively decomposed into a stem and a suffix. On the other hand, an oddball response only in Conditions 1 and 3 would indicate sensitivity to, respectively, stems and suffixes only when presented in an exhaustively decomposable morphological oddball (i.e., in the presence of another morpheme). Adults were administered all four conditions, while children were only administered Conditions 1 and 3, in consideration of the limited time that they could spend in the MEG room.

## Materials and methods

### Participants

We recruited 32 skilled adult readers (age range: 18–45) and 21 developing readers (enrolled in Grades 5 and 6 of the Australian school system at the time of testing). Data from four adults and four children were eventually removed from the final sample analyzed here, either for excessive head motion (greater than 5 mm for adults; greater than 11 mm for children) or due to an excessive presence of artifacts. This left us with 28 skilled adult readers (age: mean = 22.93 years, sd = 6.38 years) and 17 developing readers (age: mean = 10.59 years, sd = 0.79). Adult participants were recruited through Macquarie University and were offered course credit, where applicable, or monetary compensation. Children were recruited through *Neuronauts*, a dedicated Macquarie University portal, and their families were awarded monetary compensation for their time. Both studies were approved by the Macquarie University Human Research Ethics Committee. All participants were native English speakers and right-handed; none reported neurological or developmental issues, language difficulties, or claustrophobia. They all had normal or corrected-to-normal (through contact lenses) vision.

### Stimuli

All conditions consisted of five 60-s trials, for adults, and of six 60-s trials, for children. A within-participant block design was adopted. A manipulation-check condition (Condition 0) probing visual word identification was administered to all participants. Adults were administered four experimental conditions, while children were only administered two of these (Conditions 1 and 3).

In Condition 0, 4-letter English words (oddball stimuli) were embedded in non-pronounceable 4-consonant strings (base stimuli). The purpose of this condition was to ensure that the paradigm worked correctly, as prior FPVS research reports robust oddball responses to words embedded in nonwords (see, e.g., Lochy et al., 2015, 2016). In Condition 1, oddball stimuli were pseudowords made up of a real English stem and a real English suffix (e.g., *softity*), which were embedded in pseudowords made up of a nonstem and a real English suffix (e.g., *trumess*). In Condition 2, pseudowords made up of a real stem and a nonsuffix (e.g., *softert*) were used as oddballs and embedded in pseudowords made up of a nonstem and a nonsuffix (e.g., *trumust*). In Condition 3, oddball pseudowords were made up of a real stem and a real suffix (e.g., *softity*) and were embedded in pseudowords made up of a real stem and a nonsuffix (e.g., *stopust*). Lastly, in Condition 4, pseudowords made up of a nonstem and a nonsuffix (e.g., *terpity*) were embedded in pseudowords made up of a nonstem and a suffix (e.g., *trumust*). Sequence examples for each condition are reported in Figure 1.

Contrasts in each condition were set in order to tap into stem or suffix identification. Specifically, an oddball response in Condition 1 (and 2, in the adult sample only) would index stem identification, and in Condition 3 (and 4, in the adult sample only) it would index suffix identification. The administration of two additional conditions (2 and 4) to the adult participants was intended to shed light on the role of context for the identification of morphemes—that is, whether a robust response to oddballs was present only when they could be fully broken down into the two constituent morphemes (Conditions 1 and 3), or whether morphemes would also be successfully identified when oddballs featured only one morphemic constituent (Conditions 2 and 4).

In the version of the experiment with adult readers, stimuli were composed of 12 items for each type: stems, nonstems, suffixes, and nonsuffixes. Nonstems and nonsuffixes were created from the set of existing stems and existing suffixes, while keeping the same length, Consonant-Vowel structure, and minimising orthographic overlap with existing words (e.g., *terp* was created as a nonstem from *soft*, *ert* was created as a nonsuffix from *ity*). Stem and nonstems were 4 letters in length, while suffixes and nonsuffixes were 3-letter long. The 12 nonsuffixes were non-morphemic endings attested in English. Each set of (non)stems and (non)suffixes was divided into two subsets of 6 items; stimuli were then obtained by combining each element in one subset with each element of another. This procedure generated 72 unique combinations (6 items in the first set, times 6 items in the second set, times 2 subsets) of each type (stem + suffix, nonstem + suffix, stem + nonsuffix, nonstem + nonsuffix), yielding a total of 288 unique stimuli.

In the developmental version of the experiment, the building blocks were reduced to 6, a subset of those used for skilled adult readers (Non)stems and (non)suffixes were combined by groups of 3, to obtain 18 (3*3*2) unique combinations of each type (stem + suffix, nonstem + suffix, stem + nonsuffix), yielding a total of 54 unique stimuli.

All building blocks (stems, nonstems, suffixes, and nonsuffixes) are reported in Table 1. Statistics for stems and suffixes were obtained from two different linguistic databases: SUBTLEX-UK (Van Heuven et al., 2014) and MorphoLex (Sánchez-Gutiérrez et al., 2018). Specifically, while the former frequency database is particularly relevant for its size (over 160,000 types and 200 million tokens from English television show subtitles), the latter resource is a rich morphologically tagged database for English, allowing the extraction of metrics related to the use of items as morphemes in the language.

**Table 1:** Unique stems, nonstems, suffixes, and nonsuffixes combined to generate nonword stimuli, and the respective OLD20 statistics. All listed items were used to construct stimuli in the study with adults, while a subset (underlined items) was used to construct stimuli for the study with children. The full sets of stimuli are reported in the Supplementary Tables S1, S2.

**Stem**	**Nonstem**	**Suffix**	**Nonsuffix**	**OLD20 stem**	**OLD20 nonstem**	**OLD20 suffix**	**OLD20 nonsuffix**
help	josk	ity	ert	1.00	1.55	1.00	1.00
soft	terp	ive	une	1.00	1.00	1.00	1.00
last	firn	ory	ute	1.00	1.00	1.00	1.00
ship	bron	ure	int	1.00	1.00	1.00	1.00
stop	trum	ous	ald	1.00	1.00	1.00	1.00
hold	burk	ise	ere	1.00	1.00	1.00	1.00
park	molp	ful	sal	1.00	1.40	1.00	1.00
jump	lort	ist	arn	1.00	1.00	1.00	1.00
town	bemp	ite	ene	1.00	1.40	1.00	1.00
bird	jelt	ish	ult	1.00	1.00	1.00	1.00
farm	culp	ese	oke	1.00	1.35	1.00	1.00
milk	tand	ess	ust	1.00	1.00	1.00	1.00

#### Stem selection

All selected stems were four-character long and had a CVCC or CCVC consonant-vowel structure. Here, we describe the features of the 12 stems used as constituents in the adult version of the experiment, a subset of which was used in the version with developing readers; the statistics related to the six stems used in the version for children are provided in square brackets. Database exploration, extraction, and calculation of relevant metrics were performed using R (R Core Team, 2021) within RStudio (RStudio Team, 2021). The average SUBTLEX-UK log Zipf frequency was 5.13, with a sd of 0.43 [mean: 4.93, sd: 0.28]; the average stem token frequency in MorphoLex was 155,217, with a sd of 1,44,128.60 [mean: 1,32,510, sd: 1,27,152.30], while the average stem family size in MorphoLex was 15.50, with a sd of 9.64 [mean: 22.83, sd: 8.33]. Finally, the average Levenshtein distance (OLD20; Yarkoni et al., 2008), a lexical density index based on the average distance of the 20 nearest neighbors in the lexicon, was calculated through the R package *vwr* (Keuleers, 2013) using SUBTLEX-UK, the largest resource considered here. The higher the OLD20 value of a stimulus, the lower its orthographic neighborhood. All stems had a mean OLD20 of 1 and a sd of 0 [mean: 1, sd: 0].

#### Nonstem selection

Nonstems were pseudowords generated with the same length and CV structure types as the real stems, for the items to be orthographically and phonotactically legal, while at the same time minimizing orthographic overlap with the selected stems. The mean OLD20 for our nonstem selection was 1.14, with a sd of 0.21 [mean: 1.07, sd: 0.16].

#### Suffix selection

Twelve three-letter derivational suffixes were shortlisted from the CELEX database (Baayen et al., 1993). The CV structure types of the selected suffixes were VCC, VCV, CVC, and VVC. A subset of six suffixes was used for the developmental version of the experiment. The same exploration and analysis were performed as for the above-described stem selection. The average SUBTLEX-UK log Zipf frequency was 2.41, with a sd of 0.59 [mean: 2.41, sd: 0.73]. We ensured, through MorphoLex, that all selected items were productive suffixes in the English language. The average suffix token frequency in MorphoLex was 5,14,914.40, with a sd of 4,52,230.50 [mean: 6,43,484.20, sd: 5,23,213.40], while the average suffix family size in MorphoLex was 319.25, with a sd of 226.44 [mean: 431.50, sd: 145.89]. All suffixes had a mean OLD20 of 1 and a sd of 0 [mean: 1, sd: 0].

#### Nonsuffix selection

We selected 12 three-letter clusters that occur as non-morphological endings in English, with a mean OLD20 of 1 and a sd of 0 [mean: 1, sd: 0].

#### Manipulation-check condition stimuli

For Condition 0, we selected 72 4-letter words (with various CV structure types, but always ending with a consonant) and 72 4-letter non-pronounceable consonant strings. A subset of 18 words and 18 consonant strings was used for the experiment with children. The average SUBTLEX-UK log Zipf frequency was 4.71, with a sd of 0.54 [mean: 4.91, sd: 0.54].

#### Stimuli combinations

Statistics for the stimuli used in the developmental version of the experiment, which did not feature nonstem + nonsuffix combinations, are reported in brackets. OLD20 statistics were then computed for all stimuli. Stem + suffix combinations had a mean OLD20 of 2.32 and a sd of 0.30 [mean: 2.43, sd: 0.27], stem + nonsuffix combinations had a mean OLD20 of 2.49 and a sd of 0.37 [mean: 2.55, sd: 0.43], nonstem + suffix combinations had a mean OLD20 of 2.47 and a sd of 0.32 [mean: 2.47, sd: 0.32], and nonstem + nonsuffix combinations had a mean OLD20 of 2.62 and a sd of 0.31. All unique stimuli administered to skilled adult readers can be found in the Supplementary material.

### Trial structure

Each trial comprised a 60-s stimulation sequence in which stimuli were presented *via* sinusoidal contrast modulation at 6 Hz (i.e., six stimuli per second)—each individual stimulus appeared gradually, reaching a contrast peak after 83.5 ms (for a schematic illustration, see Figure 1). Each 60-s trial thus contained 360 stimuli overall. Each oddball stimulus appeared every five items (6 Hz/5 = 1.2 Hz); therefore, the stimulation sequence in each trial included 72 oddballs and 288 base items. The oddball stimuli were unique items in the adult design, whereas in the developmental design a greater number of item repetitions was present: in each trial, every oddball was delivered a total of 4 times (18*4 = 72). The sets of stimuli were generated through pseudo-randomization, using in-house R scripts within RStudio for the adult version of the experiment, and using Mix software (Van Casteren and Davis, 2006) for the developmental version. As the process could not be entirely automated, lists were then checked and edited manually when deemed necessary, in order to prevent repetitions of the same combinations within each stimulation sequence. With both skilled and developing readers, we ensured that the same stimulus was not repeated within each 1-s of the stimulation sequence (i.e., the minimum distance between stimulus repetitions was 5). Overlayed to this stimulus sequence, a fixation cross (12 pixels) was constantly present at the center of the screen. The color of the cross changed randomly (from blue to red and vice versa), and participants were instructed to tap a button whenever they detected a color change (Lochy et al., 2015, 2016).

In the experiment with skilled readers, visual stimuli were displayed in black Courier New font, with a font size of 100 pt., within a white bounding box of 500*150 pixels. In the developmental version of the experiment, stimuli were slightly enlarged and they were displayed in black Courier New bold font, with a font size of 110 pt., within a white bounding box of 510*170 pixels. A large font size was adopted for both skilled and developing readers due to their distance from the screen. In both versions of the experiment, stimuli were displayed over a gray background.

### Procedure

Responses were recorded through a fiber-optic button box (fORP, Current Designs, Philadelphia, PA, United States). Accuracy in this task was very high for all participants (skilled adult readers: mean = 97.83%, sd = 1.84; developing readers: mean = 95.64%, sd = 4.84). This behavioral task was administered with the mere purpose of ensuring that participants engaged with the area in which the stimuli would be presented. Trials were separated by a 25-s break. The break ended with a 10-s countdown to the new trial. A 2-min break was given twice between recording blocks, to allow head location measurements to be performed; one last measurement was performed at the end of the MEG recording. Overall, the MEG testing in the MSR required 45–50 min with adults and a maximum of 30 min with children.

### Apparatus

Data were collected at the KIT-Macquarie Brain Research Laboratory (Sydney, Australia). Participants lay supine in a dimly lit and magnetically shielded room (MSR). Continuous MEG recordings were acquired using a 160-channel whole-head coaxial gradiometer system (KIT, Kanazawa Institute of Technology, Japan) at a sampling rate of 1,000 Hz, with an online bandpass filter of 0.03–200 Hz. Visual stimuli were delivered through a projector (sampling rate: 60 Hz) and mirrored onto a translucent screen mounted above the participant’s head, at a distance of approximately 110 cm. The experiment was controlled *via* a Windows desktop computer, using MATLAB 2019a (MATLAB, 2019) and Psychtoolbox (Brainard, 1997; Kleiner et al., 2007). Parallel port triggers were used to mark the beginning and end of each trial, and a photodiode was used to check the correct delivery of oddball stimuli, through a white square in the bottom right corner of the screen. Participants’ head shapes were recorded using the Polhemus FASTRAK system and digitizing pen (Colchester, VT, United States). Throughout the MEG recording session, participants wore an elastic cap with five marker coils which allowed tracking the head location relative to the MEG helmet and to measure motion over time.

### MEG data preprocessing

Data were preprocessed in MATLAB using the FieldTrip toolbox for EEG/MEG analysis (Oostenveld et al., 2011) as well as in-house functions. A lowpass filter of 100 Hz was applied; continuous MEG recordings were epoched into trials using a custom-made trial function. In trial epoching, a pre-stimulus interval and a post-stimulus interval were set in order to avoid edge artifacts. Respectively, the first two oddball cycles (i.e., the first 1.67 s of stimulation) and the last one (833 ms) were cut from each trial, resulting in trials of 58.33 s each (see Lochy et al., 2015). Recordings were then downsampled to 250 Hz. Data from eight subjects (four adults and four children) with excessive noise artifacts (one adult) or excessive movement artifacts (three adults and four children) were discarded entirely. Noisy channels were removed based on visual inspection, and channel interpolation was performed (neighbors were defined using FieldTrip functions through a triangulation method). One dataset per condition (five trials per condition for adults, six trials per condition for children) per participant was obtained.

### Frequency analysis

A very similar procedure to the one used in Lochy et al. (2015, 2016) was adopted. Each participant’s trials were averaged by condition and subjected to a Fast Fourier Transform. By calculating the square root of the sum of squares of the real and imaginary parts divided by the number of data points, power spectra were then computed for each sensor. As each epoch was 58.333 s long, the frequency resolution was 1/58.333 = 0.0171 Hz. The spectra were then normalized by dividing the mean power spectrum of each frequency bin by the mean of the surrounding 20 bins (10 on either side, excluding immediately adjacent bins), thus obtaining a signal-to-noise ratio metric (SNR). Oddball response was defined as the average SNR of the response at the oddball (1.2 Hz, precisely 1.1962 Hz as calculated in the collected datasets^1^) stimulation frequency and its corresponding first three harmonics (2.4, 3.6, 4.8 Hz, precisely 2.3924, 3.5886, 4.7848 Hz, as calculated in the collected datasets). Hence, the final dataset consisted of 22,400 data points for the adult sample (28 participants, times 5 conditions, times 160 channels) and 8,160 data points for the children sample (17 participants, times 3 conditions, times 160 channels).

## Results

### Cluster-based permutation analysis in the sensor space

The present results only pertain to sensor-level analysis, as source-level analysis could not be performed due to technical limitations. A data-driven approach to the analysis was adopted. Although the primary interest is the visual identification of morphemes, and the present paradigm emphasizes quick and automatic visual access, morphological analysis might also trigger higher-level semantic processing. Therefore, we aimed at assessing the existence of any potential tagging of the oddball frequency at the whole-brain level.^2^ To this aim, we conducted a cluster-based permutation test at the sensor level (Maris and Oostenveld, 2007), adapted for FPVS-MEG datasets, which span over space (sensors), but not time. Using grand-averaged datasets per participant per condition, cluster-based permutation was performed on the power spectrum at the averaged oddball frequency and first three harmonics (see “Frequency Analysis”), across all 160 sensors. We used a within-subject design and adopted a Montecarlo method for calculating probabilities. A minimum of two neighboring channels were required for a cluster to be defined. A cluster alpha level of 0.05 was set and a one-tailed *t*-test was run (we only contemplated the hypothesis that the SNR was higher than 1). An alpha level of 0.05 was set and 5,000 randomizations were performed. With this configuration, cluster-based permutation was run against an array of ones, representing the noise level in each channel (i.e., the null distribution).

**Figure 2:**
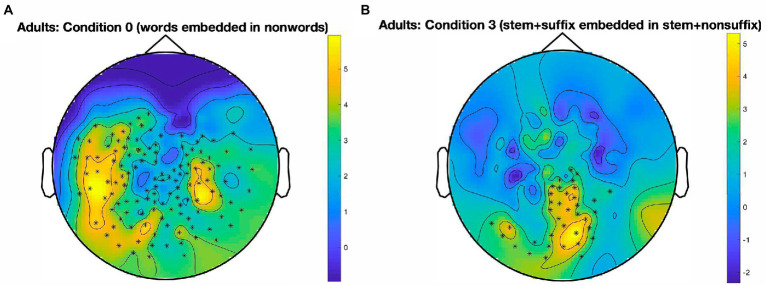
Sensor-level clusters in which a significant oddball response emerged, by condition. Data from skilled adult readers. **(A)**: Large temporo-parieto-occipital cluster (mostly left-lateralized, with a right-lateralized part) indicating widespread identification of words in nonwords, in Condition 0; *p* < 0.001, cluster alpha level = 0.05. **(B)**: Left and central occipital cluster for the identification of stem + suffix oddballs in stem + nonsuffix base stimuli, in Condition 3; *p* < 0.001, cluster alpha level = 0.05. Color bars represent SNR on a continuous scale (blue = low, yellow = high). Condition 0: *words* in *nonwords* (e.g., *roll* in *kltq*); Condition 3: *stem + suffix* in *stem + nonsuffix* (*softity* in *terpert*).

The results for the adult skilled readers are illustrated in Figure 2. In Condition 0, which taps into whole-word identification, we found one large cluster essentially encompassing the whole posterior part of the scalp, with a peak in the left hemisphere [*t*(27) = 416.46, *p* < 0.001, panel A]. A cluster also emerged for Condition 3, which probes suffix identification in the presence of a stem [*t*(27) = 113.02, *p* < 0.001, panel B]. This cluster is much smaller than in Condition 0 and extends along the midline from the vertex to the back of the brain, and then along the left ventral stream. No other significant clusters emerged, there was no robust response to the oddball stimuli in Condition 1 (designed to track stems in the presence of affixes), Condition 2 (stems in the absence of affixes), and Condition 4 (suffixes in the absence of stems).

**Figure 3:**
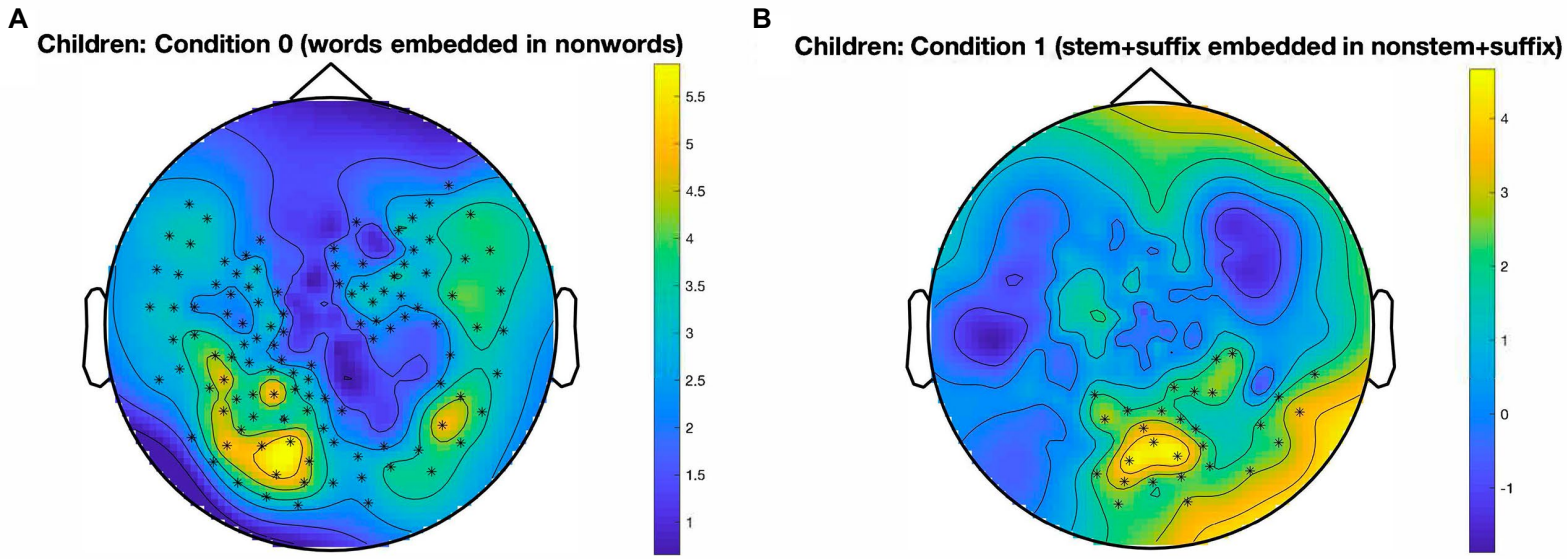
Clusters in which a significant oddball response emerged, by condition. Data from developing readers. **(A)**: Temporo-parieto-occipital cluster, largely left-lateralized, for the identification of words in nonwords, in Condition 0; *p* < 0.001, cluster alpha level = 0.05. **(B)**: Central occipital cluster for the identification of stem + suffix oddballs in nonstem + suffix base stimuli, in Condition 1; *p* = 0.007, cluster alpha level = 0.05. Color bars represent SNR on a continuous scale (blue = low, yellow = high). Condition 0: *words* in *nonwords* (*roll* in *kltq*); Condition 1: *stem + suffix* in *nonstem + suffix* (*softity* in *terpity*).

The results for the developing readers are illustrated in Figure 3. For Condition 0, a cluster emerged [*t*(16) = 347.17, *p* < 0.001, panel A] which is largely left-lateralized and extends over temporo-parieto-occipital sensors. Furthermore, an occipital cluster, mostly located around the midline, emerged in Condition 1 [*t*(16) = 74.90, *p* = 0.007, panel B], in which stem identification in the presence of suffixes is tracked.

## Discussion

Using an FPVS-oddball paradigm paired with MEG recordings, the current study sought to examine the automatic identification of morphemes in pseudowords, within a group of developing readers and a group of skilled adult readers of English. Our design also included a manipulation-check condition, that was administered to tap into whole word identification (word oddballs embedded in consonant letter strings), serving as a benchmark for our paradigm. This condition confirmed that the FPVS technique, which has already been quite extensively paired with EEG recordings, can also be successfully employed in MEG studies investigating visual word identification. A cluster-based permutation analysis at the sensor level revealed a large temporo-parieto-occipital cluster at the oddball frequency, that was present in both children and adults. As expected and consistent with previous FPVS studies using psycholinguistic material (e.g., Lochy et al., 2015, 2016), this cluster peaked predominantly in the left hemisphere for both reader groups. Interestingly, however, the cluster spread more anteriorly in adult readers. This could be taken to suggest that even automatic and implicit word identification might trigger full processing, possibly up to the semantic stage.

Although direct parallels with the studies by Lochy et al. (2015, 2016) are not fully warranted due to differences between EEG and MEG with respect to the localization of FPVS responses (see Hauk et al., 2021), one could tentatively explain the more widespread word identification response that we observe here (relative to a localized orthographic response in an occipito-temporo-parietal region) as stemming from two important differences between our studies and Lochy and colleagues’. First, in the experiment with skilled adult participants, we adopted a lower stimulation frequency (6 Hz, vs. 10 Hz in Lochy et al., 2015), resulting in a longer presentation time for each stimulus (167 ms vs. 100 ms). Second, in the developmental version of the experiment, our participants were already quite experienced readers (fifth and sixth graders) compared to the preschooler sample of Lochy et al. (2016).

Remarkably, word identification responses were similar across our developing and adult participants. These findings suggest that fifth and sixth graders, with just a few years of reading instructions, have already built up a highly sophisticated visual word identification system, roughly comparable to that of adults, when it comes to automatic and implicit detection of real words.

In relation to the core research question of the present study, whether automatic identification of morphemes emerges in complex pseudowords, the results revealed several intriguing differences between adults and children. With respect to the identification of morphemes, stems were more reliably identified by developing readers, whereas skilled readers showed sensitivity to suffixes. Suffixes represent salient units in the language, both from a semantic (as they convey systematic meaning) and from a perceptual and orthographic (as frequent chunks) point of view (Lelonkiewicz et al., 2020). Solid signs of suffix identification emerged only in the context of exhaustively decomposable complex pseudowords, which suggests that the process is not simply a catch of frequent and salient units, but involves a comprehensive (morphological) analysis of the whole string. This result aligns with the wealth of studies showing that our cognitive system heavily relies on morphology during reading and visual word identification (e.g., Rastle et al., 2000; Marslen-Wilson and Tyler, 2007; Amenta and Crepaldi, 2012; Whiting et al., 2015; Leminen et al., 2019; Beyersmann et al., 2021a). Interestingly, this finding highlights one main limitation of many current models of the visual identification of complex words (e.g., Crepaldi et al., 2010; Taft and Nguyen-Hoan, 2010; Grainger and Ziegler, 2011). These models are fundamentally based on a spreading activation mechanism, and therefore would all predict that a stem is activated any time their constituting letters are present in the input. There is no plausible computational mechanism in those models that would explain how the presence of a suffix vs. a non-suffix might trigger vs. kill the activation of a stem representation.

Our pattern of results is also in tune with findings by Beyersmann et al. (2021b), who provided evidence for a greater steady-state visually evoked potential (SSVEP) magnitude for suffixes, compared to non-suffixes. Such activation boost is taken as an index of an additional semantic feedback mechanism, beyond morphological decomposition, which would instead be sufficient for the identification of stems.

In our developmental sample, we found evidence for stem identification, again, in exhaustively decomposable stimuli (i.e., made up of a real stem and a suffix), suggesting that children in Grades 5–6 have already developed an automatic morpheme identification system, albeit not adult-like. There may be two reasons for the presence of a stem (but not suffix) response. First, stems are often encountered as whole words in English (see, e.g., Grainger and Beyersmann, 2017, 2021); from this point of view, they might be even more perceptually salient than suffixes, given that the surrounding blank spaces might serve as “chunking cues” that help the system identify these items as important functional units. Second, stems are more informative about word identity, allowing to narrow the lexical and semantic interpretation of a word more than a suffix does *per se*. For example, upon encountering *dark-*, a reader can reliably predict the general meaning of the rest of that word; instead, many different words end in *-ness*.

In line with this, Grainger and Beyersmann (2017) suggested that what is typically interpreted as morpho-orthographic processing may in fact reflect a mechanism of embedded word/stem identification that is not, *per se*, genuinely morphological, i.e., it would operate independently of the presence of an affix. This account matches with the recent observation that when lexical competition is partialled out in priming experiments—that is, when pseudowords are used—affixed and non-affixed primes provide the same amount of facilitation (*farmald-FARM* = *farmness-FARM*). Note, however, that this hypothesis could not be fully tested in our developmental sample, as the oddball stimuli in the two experimental conditions administered to children comprised only fully decomposable pseudowords. Moreover, the adult data seems to challenge this assumption, as clear signs of sensitivity to the stems only emerged in the presence of a suffix; this might be due to the intrinsic differences between the priming tasks that contributed most of the experimental basis for Grainger’s and Beyersmann’s model, and the paradigm we employed here.

Overall, the present findings can be interpreted as corroborating SSVEP evidence by Beyersmann et al. (2021a), where, on the one hand, rapid stem identification was facilitated by the presence of a suffix (or pseudo-suffix), and, on the other, suffixed words received an activation boost relative to non-suffixed ones. This is traced back to the same mechanism: the activation of embedded stems. The observed neural response to stems in children and to suffixes in adults suggests that sensitivity to morphemes differs across reading development, with stems being identified as salient units by the developing reading system (see also Grainger and Beyersmann, 2017, 2021), and suffixes acquiring saliency in a more mature system, due to the higher frequency with which they are encountered in words.

With some caution against making assumptions about potential neural sources, the fact that a response is elicited by morphemes in sensors that span over occipital regions suggests that suffixes are likely processed as visual units, at least at this stage of processing. This aligns with theories positing the existence of a level of morphological analysis that is mostly based on form (e.g., Crepaldi et al., 2010; Grainger and Ziegler, 2011; Xu and Taft, 2014). At this level of analysis, morphemes are primarily seen as frequent, statistically associated clusters of letters, perhaps not so different from what happens in other domains of vision (e.g., Vidal et al., 2021). It is well known that neural circuitry in the ventral stream is particularly apt at finding regularities in the co-occurrence of lower-level units, to then build higher-level representations that exploit such regularities (e.g., Dehaene et al., 2005; Tkačik et al., 2010). This property is particularly prominent in the domain of visual word identification, which is characterized by lower-level units (i.e., letters) that bind together higher-level objects (i.e., morphemes and words). In this context, it should not be surprising that morphemes are captured as chunks of strongly associated letters.

Experimental evidence in support of a view of visual word identification as mostly relying on the detection of (also morphological) regularities is growing. For example, Chetail (2017) asked participants to familiarize themselves with an artificial lexicon made up of pseudo-characters. The lexicon was such that some bigrams were particularly frequent; when participants were involved in a wordlikeness task with entirely novel stimuli, those that contained the frequent bigrams were judged as more word-like. So, even in a completely unfamiliar novel lexicon, made up of completely unfamiliar pseudo-characters, a few minutes of exposure were sufficient for participants to develop sensitivity to small clusters of particularly high frequency. With a similar design and experiment, Lelonkiewicz et al. (2020) were able to reproduce effects that emerged in morphological pseudowords (e.g., Taft and Forster, 1975; Crepaldi et al., 2010) with an artificial lexicon that was entirely devoid of any phonological or semantic ties, that is, a set of purely visual, non-linguistic entities made up of sequences of pseudo-characters. These data suggest that at least part of the morphological effects that we observe with genuine linguistic material can be reproduced in purely visual, non-linguistic systems.

It is less clear why in the children’s data (with respect to stem identification), and partially in the adults’ data (with respect to suffix identification), a cluster for morpheme identification emerged centrally in occipital sensors. Further FPVS-MEG investigations of the neural source(s) of morpheme identification response would ideally complement the sensor-level findings that we reported. A cautious explanation for the largely central cluster for stem identification observed in the developing readers is that it might reflect a type of processing which is less specific to morpho-orthographic units, perhaps suggestive of a more general lexical/semantic response. This would align with accounts according to which, along reading development, morpho-semantic processing matures earlier than morpho-orthographic processing, which is hypothesized to emerge only at the last stages of reading development (Grainger and Beyersmann, 2017; Beyersmann and Grainger, 2022), and to still be maturing during adolescence (Dawson et al., 2018). Alternatively, the perceptual and automatic nature of a paradigm like FPVS might have boosted those components of morpheme identification that are not specifically linguistic, but more generally visual in nature. This would again be in line with recent evidence showing that several aspects of orthographic and morphological processing can be replicated with exclusively visual material that shares the same statistical features of real language (e.g., Chetail, 2017; Lelonkiewicz et al., 2020; Vidal et al., 2021).

## Conclusion

In the present FPVS-MEG study, we showed that in Grade 5-6 children sensitivity to morphological structure, albeit not adult-like, has already sufficiently matured to be captured through an implicit, behavior-free paradigm such as FPVS. Moreover, the present results suggest that morpheme identification is stronger in strings that can be exhaustively decomposed into their constituent morphemes (i.e., when both a real stem and a real suffix are present). Signs of this identification process appeared in sensors that morphological identification as a predominantly visual process, and thus potentially linked to language-agnostic, statistical learning mechanisms (e.g., Rastle and Davis, 2003; Crepaldi et al., 2010; Lelonkiewicz et al., 2020; Vidal et al., 2021). Additionally, our findings make a methodological contribution by providing a further demonstration that the FPVS paradigm can be employed to investigate even more fine-grained processes of visual world recognition than previously explored in the literature.

## Data availability statement

Analysis scripts and data can be accessed through the project’s OSF repository: https://osf.io/ns93h/.

## Ethics statement

The studies involving human participants were reviewed and approved by the Ethics Committee of Macquarie University, Sydney, NSW, Australia. Written informed consent to participate in this study was provided by the participants or, in the case of underage participants, by a parent/legal guardian/next of kin.

## Author contributions

The study was conceptualized and designed by DC, EB, MK, and VP, with input from AC. Experimental stimuli and lists were created by VP, in collaboration with DC, EB, MK, and with input from AC. Participant recruitment and MEG data collection were performed by VP. MG data were preprocessed and analyzed by VP, under the guidance of PS, DC, EB, and MK. An initial draft of the manuscript was written by VP, with input from MK, DC, and EB. All authors provided feedback, contributed to the article, and approved the submitted version.

## Funding

This research was funded by the European Research Council (ERC) Starting Grant no. 679010 (STATLEARN), awarded to DC.

## Conflict of interest

The authors declare that the research was conducted in the absence of any commercial or financial relationships that could be construed as a potential conflict of interest.

## Publisher’s note

All claims expressed in this article are solely those of the authors and do not necessarily represent those of their affiliated organizations, or those of the publisher, the editors and the reviewers. Any product that may be evaluated in this article, or claim that may be made by its manufacturer, is not guaranteed or endorsed by the publisher.

## Footnotes

^1^This calculation was performed by means of a custom Matlab function which calculates the observed frequency of stimulus delivery, averaged across participants.

^2^As a sanity check of the effectiveness of the visual stimulation, we furthermore ensured that all participants displayed neural entrainment at the base stimulation frequency (6 Hz). See relevant section in the Supplementary material.
