# Supplementary material for: Automatic morpheme identification across development: Magnetoencephalography (MEG) evidence from fast periodic visual stimulation
Source: Front Psychol. 2022 Sep 7;13:932952. doi: 10.3389/fpsyg.2022.932952 (PMC9491359; doi:10.3389/fpsyg.2022.932952)
Supplement: Supplementary file 1 [file Data_Sheet_1.docx]

# *Supplementary Material*

**1 Table S1**

The table illustrates the complete set of unique combinations of stems/nonstems and suffixes/nonsuffixes (N=288) used as experimental stimuli for the study with skilled readers.

The table header is to be read as follows: “(Non)stem” refers to the stem (or nonstem) used as first constituent of the pseudoword. “(Non)suffix” refers to the suffix (or nonsuffix) used as second constituent of the pseudoword. “(Non)stem ID” and “(Non)suffix ID” are the unique identifiers for (non)stems and (non)suffixes, used for the creation of semi-randomized lists. “Stimulus” is the pseudoword combination used as stimulus. “Stimulus ID” is the unique identifier of the stimulus, resulting from the two constituents’ IDs and from a description of the combination used for the stimulus. “Global OLD20” refers to the stimulus’ OLD20 metric, as obtained from SUBTLEX-UK (Van Heuven et al., 2014), using the OLD20 function from the R package *vwr* (Keuleers, 2013).

**Color code**: light blue = stem+suffix combinations (N=72); light green = stem+nonsuffix combinations (N=72); yellow = nonstem+suffix combinations (N=72); orange = nonstem+nonsuffix combinations (N=72).

| **(Non)stem** | **(Non)suffix** | **(Non)stem ID** | **(Non)suffix ID** | **Stimulus** | **Stimulus ID** | **Global OLD20** |
| --- | --- | --- | --- | --- | --- | --- |
| soft | ity | 2 | 1 | softity | 0201_StemSuffix | 2.50 |
| ship | ity | 4 | 1 | shipity | 0401_StemSuffix | 2.25 |
| stop | ity | 5 | 1 | stopity | 0501_StemSuffix | 2.65 |
| hold | ity | 6 | 1 | holdity | 0601_StemSuffix | 2.30 |
| jump | ity | 8 | 1 | jumpity | 0801_StemSuffix | 2.20 |
| farm | ity | 11 | 1 | farmity | 1101_StemSuffix | 2.10 |
| soft | ous | 2 | 5 | softous | 0205_StemSuffix | 2.75 |
| ship | ous | 4 | 5 | shipous | 0405_StemSuffix | 2.85 |
| stop | ous | 5 | 5 | stopous | 0505_StemSuffix | 2.35 |
| hold | ous | 6 | 5 | holdous | 0605_StemSuffix | 2.45 |
| jump | ous | 8 | 5 | jumpous | 0805_StemSuffix | 2.60 |
| farm | ous | 11 | 5 | farmous | 1105_StemSuffix | 1.95 |
| soft | ful | 2 | 7 | softful | 0207_StemSuffix | 2.85 |
| ship | ful | 4 | 7 | shipful | 0407_StemSuffix | 2.45 |
| stop | ful | 5 | 7 | stopful | 0507_StemSuffix | 2.75 |
| hold | ful | 6 | 7 | holdful | 0607_StemSuffix | 2.60 |
| jump | ful | 8 | 7 | jumpful | 0807_StemSuffix | 2.95 |
| farm | ful | 11 | 7 | farmful | 1107_StemSuffix | 2.40 |
| soft | ite | 2 | 9 | softite | 0209_StemSuffix | 2.50 |
| ship | ite | 4 | 9 | shipite | 0409_StemSuffix | 2.45 |
| stop | ite | 5 | 9 | stopite | 0509_StemSuffix | 2.30 |
| hold | ite | 6 | 9 | holdite | 0609_StemSuffix | 2.05 |
| jump | ite | 8 | 9 | jumpite | 0809_StemSuffix | 2.55 |
| farm | ite | 11 | 9 | farmite | 1109_StemSuffix | 1.95 |
| **(Non)stem** | **(Non)suffix** | **(Non)stem ID** | **(Non)suffix ID** | **Stimulus** | **Stimulus ID** | **Global OLD20** |
| soft | ese | 2 | 11 | softese | 0211_StemSuffix | 2.20 |
| ship | ese | 4 | 11 | shipese | 0411_StemSuffix | 2.45 |
| stop | ese | 5 | 11 | stopese | 0511_StemSuffix | 2.40 |
| hold | ese | 6 | 11 | holdese | 0611_StemSuffix | 2.20 |
| jump | ese | 8 | 11 | jumpese | 0811_StemSuffix | 2.75 |
| farm | ese | 11 | 11 | farmese | 1111_StemSuffix | 1.90 |
| soft | ess | 2 | 12 | softess | 0212_StemSuffix | 2.10 |
| ship | ess | 4 | 12 | shipess | 0412_StemSuffix | 2.00 |
| stop | ess | 5 | 12 | stopess | 0512_StemSuffix | 1.95 |
| hold | ess | 6 | 12 | holdess | 0612_StemSuffix | 1.90 |
| jump | ess | 8 | 12 | jumpess | 0812_StemSuffix | 2.55 |
| farm | ess | 11 | 12 | farmess | 1112_StemSuffix | 1.95 |
| help | ive | 1 | 2 | helpive | 0102_StemSuffix | 2.55 |
| last | ive | 3 | 2 | lastive | 0302_StemSuffix | 1.90 |
| park | ive | 7 | 2 | parkive | 0702_StemSuffix | 1.95 |
| town | ive | 9 | 2 | townive | 0902_StemSuffix | 2.45 |
| bird | ive | 10 | 2 | birdive | 1002_StemSuffix | 2.40 |
| milk | ive | 12 | 2 | milkive | 1202_StemSuffix | 2.25 |
| help | ory | 1 | 3 | helpory | 0103_StemSuffix | 2.75 |
| last | ory | 3 | 3 | lastory | 0303_StemSuffix | 1.95 |
| park | ory | 7 | 3 | parkory | 0703_StemSuffix | 2.20 |
| town | ory | 9 | 3 | townory | 0903_StemSuffix | 2.75 |
| bird | ory | 10 | 3 | birdory | 1003_StemSuffix | 2.75 |
| milk | ory | 12 | 3 | milkory | 1203_StemSuffix | 2.20 |
| **(Non)stem** | **(Non)suffix** | **(Non)stem ID** | **(Non)suffix ID** | **Stimulus** | **Stimulus ID** | **Global OLD20** |
| help | ure | 1 | 4 | helpure | 0104_StemSuffix | 2.80 |
| last | ure | 3 | 4 | lasture | 0304_StemSuffix | 1.95 |
| park | ure | 7 | 4 | parkure | 0704_StemSuffix | 2.35 |
| town | ure | 9 | 4 | townure | 0904_StemSuffix | 2.60 |
| bird | ure | 10 | 4 | birdure | 1004_StemSuffix | 2.65 |
| milk | ure | 12 | 4 | milkure | 1204_StemSuffix | 2.60 |
| help | ise | 1 | 6 | helpise | 0106_StemSuffix | 2.35 |
| last | ise | 3 | 6 | lastise | 0306_StemSuffix | 2.00 |
| park | ise | 7 | 6 | parkise | 0706_StemSuffix | 1.90 |
| town | ise | 9 | 6 | townise | 0906_StemSuffix | 2.30 |
| bird | ise | 10 | 6 | birdise | 1006_StemSuffix | 2.25 |
| milk | ise | 12 | 6 | milkise | 1206_StemSuffix | 2.05 |
| help | ist | 1 | 8 | helpist | 0108_StemSuffix | 2.35 |
| last | ist | 3 | 8 | lastist | 0308_StemSuffix | 1.90 |
| park | ist | 7 | 8 | parkist | 0708_StemSuffix | 1.95 |
| town | ist | 9 | 8 | townist | 0908_StemSuffix | 2.50 |
| bird | ist | 10 | 8 | birdist | 1008_StemSuffix | 2.40 |
| milk | ist | 12 | 8 | milkist | 1208_StemSuffix | 2.45 |
| help | ish | 1 | 10 | helpish | 0110_StemSuffix | 2.15 |
| last | ish | 3 | 10 | lastish | 0310_StemSuffix | 2.00 |
| park | ish | 7 | 10 | parkish | 0710_StemSuffix | 1.85 |
| town | ish | 9 | 10 | townish | 0910_StemSuffix | 2.05 |
| bird | ish | 10 | 10 | birdish | 1010_StemSuffix | 2.10 |
| milk | ish | 12 | 10 | milkish | 1210_StemSuffix | 2.25 |
| **(Non)stem** | **(Non)suffix** | **(Non)stem ID** | **(Non)suffix ID** | **Stimulus** | **Stimulus ID** | **Global OLD20** |
| soft | ert | 2 | 1 | softert | 0201_StemNonsuffix | 2.25 |
| ship | ert | 4 | 1 | shipert | 0401_StemNonsuffix | 2.00 |
| stop | ert | 5 | 1 | stopert | 0501_StemNonsuffix | 2.00 |
| hold | ert | 6 | 1 | holdert | 0601_StemNonsuffix | 1.90 |
| jump | ert | 8 | 1 | jumpert | 0801_StemNonsuffix | 1.95 |
| farm | ert | 11 | 1 | farmert | 1101_StemNonsuffix | 1.80 |
| soft | ald | 2 | 5 | softald | 0205_StemNonsuffix | 2.90 |
| ship | ald | 4 | 5 | shipald | 0405_StemNonsuffix | 2.80 |
| stop | ald | 5 | 5 | stopald | 0505_StemNonsuffix | 2.85 |
| hold | ald | 6 | 5 | holdald | 0605_StemNonsuffix | 2.75 |
| jump | ald | 8 | 5 | jumpald | 0805_StemNonsuffix | 2.95 |
| farm | ald | 11 | 5 | farmald | 1105_StemNonsuffix | 2.20 |
| soft | sal | 2 | 7 | softsal | 0207_StemNonsuffix | 2.90 |
| ship | sal | 4 | 7 | shipsal | 0407_StemNonsuffix | 2.70 |
| stop | sal | 5 | 7 | stopsal | 0507_StemNonsuffix | 2.60 |
| hold | sal | 6 | 7 | holdsal | 0607_StemNonsuffix | 2.80 |
| jump | sal | 8 | 7 | jumpsal | 0807_StemNonsuffix | 2.90 |
| farm | sal | 11 | 7 | farmsal | 1107_StemNonsuffix | 2.75 |
| soft | ene | 2 | 9 | softene | 0209_StemNonsuffix | 2.05 |
| ship | ene | 4 | 9 | shipene | 0409_StemNonsuffix | 2.55 |
| stop | ene | 5 | 9 | stopene | 0509_StemNonsuffix | 2.60 |
| hold | ene | 6 | 9 | holdene | 0609_StemNonsuffix | 1.95 |
| jump | ene | 8 | 9 | jumpene | 0809_StemNonsuffix | 2.65 |
| farm | ene | 11 | 9 | farmene | 1109_StemNonsuffix | 1.95 |
| **(Non)stem** | **(Non)suffix** | **(Non)stem ID** | **(Non)suffix ID** | **Stimulus** | **Stimulus ID** | **Global OLD20** |
| soft | oke | 2 | 11 | softoke | 0211_StemNonsuffix | 2.75 |
| ship | oke | 4 | 11 | shipoke | 0411_StemNonsuffix | 2.75 |
| stop | oke | 5 | 11 | stopoke | 0511_StemNonsuffix | 2.30 |
| hold | oke | 6 | 11 | holdoke | 0611_StemNonsuffix | 2.70 |
| jump | oke | 8 | 11 | jumpoke | 0811_StemNonsuffix | 2.95 |
| farm | oke | 11 | 11 | farmoke | 1111_StemNonsuffix | 2.60 |
| soft | ust | 2 | 12 | softust | 0212_StemNonsuffix | 2.75 |
| ship | ust | 4 | 12 | shipust | 0412_StemNonsuffix | 2.85 |
| stop | ust | 5 | 12 | stopust | 0512_StemNonsuffix | 2.70 |
| hold | ust | 6 | 12 | holdust | 0612_StemNonsuffix | 2.50 |
| jump | ust | 8 | 12 | jumpust | 0812_StemNonsuffix | 2.85 |
| farm | ust | 11 | 12 | farmust | 1112_StemNonsuffix | 2.70 |
| help | une | 1 | 2 | helpune | 0102_StemNonsuffix | 2.80 |
| last | une | 3 | 2 | lastune | 0302_StemNonsuffix | 2.55 |
| park | une | 7 | 2 | parkune | 0702_StemNonsuffix | 2.50 |
| town | une | 9 | 2 | townune | 0902_StemNonsuffix | 2.75 |
| bird | une | 10 | 2 | birdune | 1002_StemNonsuffix | 2.65 |
| milk | une | 12 | 2 | milkune | 1202_StemNonsuffix | 2.65 |
| help | ute | 1 | 3 | helpute | 0103_StemNonsuffix | 2.70 |
| last | ute | 3 | 3 | lastute | 0303_StemNonsuffix | 2.70 |
| park | ute | 7 | 3 | parkute | 0703_StemNonsuffix | 2.60 |
| town | ute | 9 | 3 | townute | 0903_StemNonsuffix | 2.80 |
| bird | ute | 10 | 3 | birdute | 1003_StemNonsuffix | 2.65 |
| milk | ute | 12 | 3 | milkute | 1203_StemNonsuffix | 2.85 |
| **(Non)stem** | **(Non)suffix** | **(Non)stem ID** | **(Non)suffix ID** | **Stimulus** | **Stimulus ID** | **Global OLD20** |
| help | int | 1 | 4 | helpint | 0104_StemNonsuffix | 2.25 |
| last | int | 3 | 4 | lastint | 0304_StemNonsuffix | 1.90 |
| park | int | 7 | 4 | parkint | 0704_StemNonsuffix | 1.85 |
| town | int | 9 | 4 | townint | 0904_StemNonsuffix | 2.40 |
| bird | int | 10 | 4 | birdint | 1004_StemNonsuffix | 1.95 |
| milk | int | 12 | 4 | milkint | 1204_StemNonsuffix | 2.05 |
| help | ere | 1 | 6 | helpere | 0106_StemNonsuffix | 2.20 |
| last | ere | 3 | 6 | lastere | 0306_StemNonsuffix | 1.95 |
| park | ere | 7 | 6 | parkere | 0706_StemNonsuffix | 1.90 |
| town | ere | 9 | 6 | townere | 0906_StemNonsuffix | 1.90 |
| bird | ere | 10 | 6 | birdere | 1006_StemNonsuffix | 1.90 |
| milk | ere | 12 | 6 | milkere | 1206_StemNonsuffix | 1.90 |
| help | arn | 1 | 8 | helparn | 0108_StemNonsuffix | 2.55 |
| last | arn | 3 | 8 | lastarn | 0308_StemNonsuffix | 2.05 |
| park | arn | 7 | 8 | parkarn | 0708_StemNonsuffix | 2.15 |
| town | arn | 9 | 8 | townarn | 0908_StemNonsuffix | 2.80 |
| bird | arn | 10 | 8 | birdarn | 1008_StemNonsuffix | 2.60 |
| milk | arn | 12 | 8 | milkarn | 1208_StemNonsuffix | 2.30 |
| help | ult | 1 | 10 | helpult | 0110_StemNonsuffix | 2.90 |
| last | ult | 3 | 10 | lastult | 0310_StemNonsuffix | 2.90 |
| park | ult | 7 | 10 | parkult | 0710_StemNonsuffix | 2.80 |
| town | ult | 9 | 10 | townult | 0910_StemNonsuffix | 2.90 |
| bird | ult | 10 | 10 | birdult | 1010_StemNonsuffix | 2.95 |
| milk | ult | 12 | 10 | milkult | 1210_StemNonsuffix | 3.00 |
| **(Non)stem** | **(Non)suffix** | **(Non)stem ID** | **(Non)suffix ID** | **Stimulus** | **Stimulus ID** | **Global OLD20** |
| terp | ity | 2 | 1 | terpity | 0201_NonstemSuffix | 2.25 |
| bron | ity | 4 | 1 | bronity | 0401_NonstemSuffix | 2.45 |
| trum | ity | 5 | 1 | trumity | 0501_NonstemSuffix | 2.50 |
| burk | ity | 6 | 1 | burkity | 0601_NonstemSuffix | 2.40 |
| lort | ity | 8 | 1 | lortity | 0801_NonstemSuffix | 2.65 |
| culp | ity | 11 | 1 | culpity | 1101_NonstemSuffix | 2.55 |
| terp | ous | 2 | 5 | terpous | 0205_NonstemSuffix | 2.20 |
| bron | ous | 4 | 5 | bronous | 0405_NonstemSuffix | 2.15 |
| trum | ous | 5 | 5 | trumous | 0505_NonstemSuffix | 2.20 |
| burk | ous | 6 | 5 | burkous | 0605_NonstemSuffix | 2.40 |
| lort | ous | 8 | 5 | lortous | 0805_NonstemSuffix | 1.95 |
| culp | ous | 11 | 5 | culpous | 1105_NonstemSuffix | 2.70 |
| terp | ful | 2 | 7 | terpful | 0207_NonstemSuffix | 2.75 |
| bron | ful | 4 | 7 | bronful | 0407_NonstemSuffix | 2.75 |
| trum | ful | 5 | 7 | trumful | 0507_NonstemSuffix | 2.65 |
| burk | ful | 6 | 7 | burkful | 0607_NonstemSuffix | 2.85 |
| lort | ful | 8 | 7 | lortful | 0807_NonstemSuffix | 2.70 |
| culp | ful | 11 | 7 | culpful | 1107_NonstemSuffix | 2.70 |
| terp | ite | 2 | 9 | terpite | 0209_NonstemSuffix | 2.00 |
| bron | ite | 4 | 9 | bronite | 0409_NonstemSuffix | 1.90 |
| trum | ite | 5 | 9 | trumite | 0509_NonstemSuffix | 2.70 |
| burk | ite | 6 | 9 | burkite | 0609_NonstemSuffix | 2.35 |
| lort | ite | 8 | 9 | lortite | 0809_NonstemSuffix | 2.45 |
| culp | ite | 11 | 9 | culpite | 1109_NonstemSuffix | 2.55 |
| **(Non)stem** | **(Non)suffix** | **(Non)stem ID** | **(Non)suffix ID** | **Stimulus** | **Stimulus ID** | **Global OLD20** |
| terp | ese | 2 | 11 | terpese | 0211_NonstemSuffix | 2.30 |
| bron | ese | 4 | 11 | bronese | 0411_NonstemSuffix | 2.00 |
| trum | ese | 5 | 11 | trumese | 0511_NonstemSuffix | 2.70 |
| burk | ese | 6 | 11 | burkese | 0611_NonstemSuffix | 2.10 |
| lort | ese | 8 | 11 | lortese | 0811_NonstemSuffix | 2.45 |
| culp | ese | 11 | 11 | culpese | 1111_NonstemSuffix | 2.75 |
| terp | ess | 2 | 12 | terpess | 0212_NonstemSuffix | 2.05 |
| bron | ess | 4 | 12 | broness | 0412_NonstemSuffix | 1.95 |
| trum | ess | 5 | 12 | trumess | 0512_NonstemSuffix | 2.40 |
| burk | ess | 6 | 12 | burkess | 0612_NonstemSuffix | 1.80 |
| lort | ess | 8 | 12 | lortess | 0812_NonstemSuffix | 2.00 |
| culp | ess | 11 | 12 | culpess | 1112_NonstemSuffix | 2.70 |
| josk | ive | 1 | 2 | joskive | 0102_NonstemSuffix | 2.80 |
| firn | ive | 3 | 2 | firnive | 0302_NonstemSuffix | 2.65 |
| molp | ive | 7 | 2 | molpive | 0702_NonstemSuffix | 2.70 |
| bemp | ive | 9 | 2 | bempive | 0902_NonstemSuffix | 2.70 |
| jelt | ive | 10 | 2 | jeltive | 1002_NonstemSuffix | 2.40 |
| tand | ive | 12 | 2 | tandive | 1202_NonstemSuffix | 2.50 |
| josk | ory | 1 | 3 | joskory | 0103_NonstemSuffix | 2.95 |
| firn | ory | 3 | 3 | firnory | 0303_NonstemSuffix | 2.85 |
| molp | ory | 7 | 3 | molpory | 0703_NonstemSuffix | 2.80 |
| bemp | ory | 9 | 3 | bempory | 0903_NonstemSuffix | 2.85 |
| jelt | ory | 10 | 3 | jeltory | 1003_NonstemSuffix | 2.95 |
| tand | ory | 12 | 3 | tandory | 1203_NonstemSuffix | 2.10 |
| **(Non)stem** | **(Non)suffix** | **(Non)stem ID** | **(Non)suffix ID** | **Stimulus** | **Stimulus ID** | **Global OLD20** |
| josk | ure | 1 | 4 | joskure | 0104_NonstemSuffix | 2.85 |
| firn | ure | 3 | 4 | firnure | 0304_NonstemSuffix | 2.70 |
| molp | ure | 7 | 4 | molpure | 0704_NonstemSuffix | 2.90 |
| bemp | ure | 9 | 4 | bempure | 0904_NonstemSuffix | 2.70 |
| jelt | ure | 10 | 4 | jelture | 1004_NonstemSuffix | 2.25 |
| tand | ure | 12 | 4 | tandure | 1204_NonstemSuffix | 2.50 |
| josk | ise | 1 | 6 | joskise | 0106_NonstemSuffix | 2.90 |
| firn | ise | 3 | 6 | firnise | 0306_NonstemSuffix | 2.15 |
| molp | ise | 7 | 6 | molpise | 0706_NonstemSuffix | 2.60 |
| bemp | ise | 9 | 6 | bempise | 0906_NonstemSuffix | 2.70 |
| jelt | ise | 10 | 6 | jeltise | 1006_NonstemSuffix | 2.75 |
| tand | ise | 12 | 6 | tandise | 1206_NonstemSuffix | 2.20 |
| josk | ist | 1 | 8 | joskist | 0108_NonstemSuffix | 2.85 |
| firn | ist | 3 | 8 | firnist | 0308_NonstemSuffix | 2.25 |
| molp | ist | 7 | 8 | molpist | 0708_NonstemSuffix | 2.75 |
| bemp | ist | 9 | 8 | bempist | 0908_NonstemSuffix | 2.65 |
| jelt | ist | 10 | 8 | jeltist | 1008_NonstemSuffix | 2.60 |
| tand | ist | 12 | 8 | tandist | 1208_NonstemSuffix | 2.20 |
| josk | ish | 1 | 10 | joskish | 0110_NonstemSuffix | 2.75 |
| firn | ish | 3 | 10 | firnish | 0310_NonstemSuffix | 1.85 |
| molp | ish | 7 | 10 | molpish | 0710_NonstemSuffix | 2.10 |
| bemp | ish | 9 | 10 | bempish | 0910_NonstemSuffix | 2.60 |
| jelt | ish | 10 | 10 | jeltish | 1010_NonstemSuffix | 2.15 |
| tand | ish | 12 | 10 | tandish | 1210_NonstemSuffix | 1.90 |
| **(Non)stem** | **(Non)suffix** | **(Non)stem ID** | **(Non)suffix ID** | **Stimulus** | **Stimulus ID** | **Global OLD20** |
| terp | ert | 2 | 1 | terpert | 0201_NonstemNonsuffix | 2.25 |
| bron | ert | 4 | 1 | bronert | 0401_NonstemNonsuffix | 2.00 |
| trum | ert | 5 | 1 | trumert | 0501_NonstemNonsuffix | 2.30 |
| burk | ert | 6 | 1 | burkert | 0601_NonstemNonsuffix | 1.95 |
| lort | ert | 8 | 1 | lortert | 0801_NonstemNonsuffix | 2.25 |
| culp | ert | 11 | 1 | culpert | 1101_NonstemNonsuffix | 1.95 |
| terp | ald | 2 | 5 | terpald | 0205_NonstemNonsuffix | 2.85 |
| bron | ald | 4 | 5 | bronald | 0405_NonstemNonsuffix | 2.35 |
| trum | ald | 5 | 5 | trumald | 0505_NonstemNonsuffix | 2.85 |
| burk | ald | 6 | 5 | burkald | 0605_NonstemNonsuffix | 2.60 |
| lort | ald | 8 | 5 | lortald | 0805_NonstemNonsuffix | 2.60 |
| culp | ald | 11 | 5 | culpald | 1105_NonstemNonsuffix | 2.80 |
| terp | sal | 2 | 7 | terpsal | 0207_NonstemNonsuffix | 2.75 |
| bron | sal | 4 | 7 | bronsal | 0407_NonstemNonsuffix | 2.50 |
| trum | sal | 5 | 7 | trumsal | 0507_NonstemNonsuffix | 2.85 |
| burk | sal | 6 | 7 | burksal | 0607_NonstemNonsuffix | 2.60 |
| lort | sal | 8 | 7 | lortsal | 0807_NonstemNonsuffix | 2.70 |
| culp | sal | 11 | 7 | culpsal | 1107_NonstemNonsuffix | 2.90 |
| terp | ene | 2 | 9 | terpene | 0209_NonstemNonsuffix | 2.10 |
| bron | ene | 4 | 9 | bronene | 0409_NonstemNonsuffix | 2.40 |
| trum | ene | 5 | 9 | trumene | 0509_NonstemNonsuffix | 2.65 |
| burk | ene | 6 | 9 | burkene | 0609_NonstemNonsuffix | 2.25 |
| lort | ene | 8 | 9 | lortene | 0809_NonstemNonsuffix | 2.05 |
| culp | ene | 11 | 9 | culpene | 1109_NonstemNonsuffix | 2.65 |
| **(Non)stem** | **(Non)suffix** | **(Non)stem ID** | **(Non)suffix ID** | **Stimulus** | **Stimulus ID** | **Global OLD20** |
| terp | oke | 2 | 11 | terpoke | 0211_NonstemNonsuffix | 2.90 |
| bron | oke | 4 | 11 | bronoke | 0411_NonstemNonsuffix | 2.10 |
| trum | oke | 5 | 11 | trumoke | 0511_NonstemNonsuffix | 2.95 |
| burk | oke | 6 | 11 | burkoke | 0611_NonstemNonsuffix | 2.85 |
| lort | oke | 8 | 11 | lortoke | 0811_NonstemNonsuffix | 2.90 |
| culp | oke | 11 | 11 | culpoke | 1111_NonstemNonsuffix | 2.80 |
| terp | ust | 2 | 12 | terpust | 0212_NonstemNonsuffix | 2.75 |
| bron | ust | 4 | 12 | bronust | 0412_NonstemNonsuffix | 2.45 |
| trum | ust | 5 | 12 | trumust | 0512_NonstemNonsuffix | 2.70 |
| burk | ust | 6 | 12 | burkust | 0612_NonstemNonsuffix | 2.65 |
| lort | ust | 8 | 12 | lortust | 0812_NonstemNonsuffix | 2.80 |
| culp | ust | 11 | 12 | culpust | 1112_NonstemNonsuffix | 2.80 |
| josk | une | 1 | 2 | joskune | 0102_NonstemNonsuffix | 2.95 |
| firn | une | 3 | 2 | firnune | 0302_NonstemNonsuffix | 2.90 |
| molp | une | 7 | 2 | molpune | 0702_NonstemNonsuffix | 2.85 |
| bemp | une | 9 | 2 | bempune | 0902_NonstemNonsuffix | 2.85 |
| jelt | une | 10 | 2 | jeltune | 1002_NonstemNonsuffix | 2.70 |
| tand | une | 12 | 2 | tandune | 1202_NonstemNonsuffix | 2.85 |
| josk | ute | 1 | 3 | joskute | 0103_NonstemNonsuffix | 2.90 |
| firn | ute | 3 | 3 | firnute | 0303_NonstemNonsuffix | 2.75 |
| molp | ute | 7 | 3 | molpute | 0703_NonstemNonsuffix | 2.80 |
| bemp | ute | 9 | 3 | bempute | 0903_NonstemNonsuffix | 2.70 |
| jelt | ute | 10 | 3 | jeltute | 1003_NonstemNonsuffix | 2.85 |
| tand | ute | 12 | 3 | tandute | 1203_NonstemNonsuffix | 2.70 |
| **(Non)stem** | **(Non)suffix** | **(Non)stem ID** | **(Non)suffix ID** | **Stimulus** | **Stimulus ID** | **Global OLD20** |
| josk | int | 1 | 4 | joskint | 0104_NonstemNonsuffix | 2.40 |
| firn | int | 3 | 4 | firnint | 0304_NonstemNonsuffix | 2.50 |
| molp | int | 7 | 4 | molpint | 0704_NonstemNonsuffix | 2.65 |
| bemp | int | 9 | 4 | bempint | 0904_NonstemNonsuffix | 2.75 |
| jelt | int | 10 | 4 | jeltint | 1004_NonstemNonsuffix | 2.35 |
| tand | int | 12 | 4 | tandint | 1204_NonstemNonsuffix | 2.00 |
| josk | ere | 1 | 6 | joskere | 0106_NonstemNonsuffix | 2.85 |
| firn | ere | 3 | 6 | firnere | 0306_NonstemNonsuffix | 2.40 |
| molp | ere | 7 | 6 | molpere | 0706_NonstemNonsuffix | 2.70 |
| bemp | ere | 9 | 6 | bempere | 0906_NonstemNonsuffix | 2.15 |
| jelt | ere | 10 | 6 | jeltere | 1006_NonstemNonsuffix | 2.40 |
| tand | ere | 12 | 6 | tandere | 1206_NonstemNonsuffix | 1.95 |
| josk | arn | 1 | 8 | joskarn | 0108_NonstemNonsuffix | 2.95 |
| firn | arn | 3 | 8 | firnarn | 0308_NonstemNonsuffix | 2.80 |
| molp | arn | 7 | 8 | molparn | 0708_NonstemNonsuffix | 2.80 |
| bemp | arn | 9 | 8 | bemparn | 0908_NonstemNonsuffix | 2.80 |
| jelt | arn | 10 | 8 | jeltarn | 1008_NonstemNonsuffix | 2.90 |
| tand | arn | 12 | 8 | tandarn | 1208_NonstemNonsuffix | 2.05 |
| josk | ult | 1 | 10 | joskult | 0110_NonstemNonsuffix | 2.90 |
| firn | ult | 3 | 10 | firnult | 0310_NonstemNonsuffix | 2.95 |
| molp | ult | 7 | 10 | molpult | 0710_NonstemNonsuffix | 2.95 |
| bemp | ult | 9 | 10 | bempult | 0910_NonstemNonsuffix | 3.00 |
| jelt | ult | 10 | 10 | jeltult | 1010_NonstemNonsuffix | 3.00 |
| tand | ult | 12 | 10 | tandult | 1210_NonstemNonsuffix | 2.90 |

**2 Table S2**

The table illustrates the complete set of unique combinations of stems/nonstems and suffixes/nonsuffixes (N=54) used as experimental stimuli for the study with children.

The table header is to be read as follows: “(Non)stem” refers to the stem (or nonstem) used as first constituent of the pseudoword. “(Non)suffix” refers to the suffix (or nonsuffix) used as second constituent of the pseudoword. “(Non)stem ID” and “(Non)suffix ID” are unique identifiers for (non)stems and (non)suffixes, used for the creation of semi-randomized lists. “Stimulus” is the pseudoword combination used as stimulus. “Stimulus ID” is the unique identifier of the stimulus, resulting from the two constituents’ IDs and from a description of the combination used for the stimulus. “Global OLD20” refers to the stimulus’ OLD20 metric, as obtained from SUBTLEX-UK (Van Heuven et al., 2014), using the OLD20 function from the R package *vwr* (Keuleers, 2013).

**Color code**: light blue = stem+suffix combinations (N=18); light green = stem+nonsuffix combinations (N=18); yellow = nonstem+suffix combinations (N=18).

| **(Non)stem** | **(Non)suffix** | **(Non)stem ID** | **(Non)suffix ID** | **Stimulus** | **Stimulus ID** | **Global OLD20** |
| --- | --- | --- | --- | --- | --- | --- |
| soft | ity | 2 | 1 | softity | 0201_StemSuffix | 2.50 |
| ship | ity | 4 | 1 | shipity | 0401_StemSuffix | 2.25 |
| hold | ity | 6 | 1 | holdity | 0601_StemSuffix | 2.30 |
| soft | ous | 2 | 5 | softous | 0205_StemSuffix | 2.75 |
| ship | ous | 4 | 5 | shipous | 0405_StemSuffix | 2.85 |
| hold | ous | 6 | 5 | holdous | 0605_StemSuffix | 2.45 |
| soft | ful | 2 | 7 | softful | 0207_StemSuffix | 2.85 |
| ship | ful | 4 | 7 | shipful | 0407_StemSuffix | 2.45 |
| hold | ful | 6 | 7 | holdful | 0607_StemSuffix | 2.60 |
| town | ory | 9 | 3 | townory | 0903_StemSuffix | 2.75 |
| bird | ory | 10 | 3 | birdory | 1003_StemSuffix | 2.75 |
| milk | ory | 12 | 3 | milkory | 1203_StemSuffix | 2.20 |
| town | ise | 9 | 6 | townise | 0906_StemSuffix | 2.30 |
| bird | ise | 10 | 6 | birdise | 1006_StemSuffix | 2.25 |
| milk | ise | 12 | 6 | milkise | 1206_StemSuffix | 2.05 |
| town | ish | 9 | 10 | townish | 0910_StemSuffix | 2.05 |
| bird | ish | 10 | 10 | birdish | 1010_StemSuffix | 2.10 |
| milk | ish | 12 | 10 | milkish | 1210_StemSuffix | 2.25 |
| soft | ert | 2 | 1 | softert | 0201_StemNonsuffix | 2.25 |
| ship | ert | 4 | 1 | shipert | 0401_StemNonsuffix | 2.00 |
| hold | ert | 6 | 1 | holdert | 0601_StemNonsuffix | 1.90 |
| soft | ald | 2 | 5 | softald | 0205_StemNonsuffix | 2.90 |
| ship | ald | 4 | 5 | shipald | 0405_StemNonsuffix | 2.80 |
| hold | ald | 6 | 5 | holdald | 0605_StemNonsuffix | 2.75 |
| soft | sal | 2 | 7 | softsal | 0207_StemNonsuffix | 2.90 |
| ship | sal | 4 | 7 | shipsal | 0407_StemNonsuffix | 2.70 |
| hold | sal | 6 | 7 | holdsal | 0607_StemNonsuffix | 2.80 |
| town | ute | 9 | 3 | townute | 0903_StemNonsuffix | 2.80 |
| bird | ute | 10 | 3 | birdute | 1003_StemNonsuffix | 2.65 |
| milk | ute | 12 | 3 | milkute | 1203_StemNonsuffix | 2.85 |
| town | ere | 9 | 6 | townere | 0906_StemNonsuffix | 1.90 |
| bird | ere | 10 | 6 | birdere | 1006_StemNonsuffix | 1.90 |
| milk | ere | 12 | 6 | milkere | 1206_StemNonsuffix | 1.90 |
| town | ult | 9 | 10 | townult | 0910_StemNonsuffix | 2.90 |
| bird | ult | 10 | 10 | birdult | 1010_StemNonsuffix | 2.95 |
| milk | ult | 12 | 10 | milkult | 1210_StemNonsuffix | 3.00 |
| terp | ity | 2 | 1 | terpity | 0201_NonstemSuffix | 2.25 |
| bron | ity | 4 | 1 | bronity | 0401_NonstemSuffix | 2.45 |
| burk | ity | 6 | 1 | burkity | 0601_NonstemSuffix | 2.40 |
| terp | ous | 2 | 5 | terpous | 0205_NonstemSuffix | 2.20 |
| bron | ous | 4 | 5 | bronous | 0405_NonstemSuffix | 2.15 |
| burk | ous | 6 | 5 | burkous | 0605_NonstemSuffix | 2.40 |
| terp | ful | 2 | 7 | terpful | 0207_NonstemSuffix | 2.75 |
| bron | ful | 4 | 7 | bronful | 0407_NonstemSuffix | 2.75 |
| burk | ful | 6 | 7 | burkful | 0607_NonstemSuffix | 2.85 |
| bemp | ory | 9 | 3 | bempory | 0903_NonstemSuffix | 2.85 |
| jelt | ory | 10 | 3 | jeltory | 1003_NonstemSuffix | 2.95 |
| tand | ory | 12 | 3 | tandory | 1203_NonstemSuffix | 2.10 |
| bemp | ise | 9 | 6 | bempise | 0906_NonstemSuffix | 2.70 |
| jelt | ise | 10 | 6 | jeltise | 1006_NonstemSuffix | 2.75 |
| tand | ise | 12 | 6 | tandise | 1206_NonstemSuffix | 2.20 |
| bemp | ish | 9 | 10 | bempish | 0910_NonstemSuffix | 2.60 |
| jelt | ish | 10 | 10 | jeltish | 1010_NonstemSuffix | 2.15 |
| tand | ish | 12 | 10 | tandish | 1210_NonstemSuffix | 1.90 |

**3 Additional Analyses – Base Frequency (6 Hz) Neural Entrainment**

**Neural entrainment at 6 Hz**

To ensure that entrainment had occurred across participants, we examined whether all participants showed evidence for neural entrainment at the base stimulation frequency, namely at 6 Hz (precisely 5.9807 Hz, obtained through a calculation of the average observed stimulation frequency in the collected datasets). Regions of interest were defined as a set of 10 channels each, based on a visual inspection of the scalp topography of the 6 Hz response, both in children and adults, in all conditions (See Figures S1 and S2, respectively). One sample *t-*tests were then used to test the significance of the neural responses in each predefined region of interest and each condition.

*Skilled adult readers.* Based on visual inspection of the scalp topography (Figure S1), we defined a sensor-level ROI which spanned across the following 10 parieto-occipital sensors: 134, 137, 139, 140, 145, 150, 151, 152, 153, 154.

At 6 Hz, results revealed a significant response, in all conditions (see plot in Figure S2):

Condition 0 (*t*(27) = 10.189, *p* < .001);

Condition 1 (*t*(27) = 9.439, *p* <.001);

Condition 2 (*t*(27) = 10.358, *p* <.001);

Condition 3 (*t*(27) = 10.974, *p* <.001);

Condition 4 (*t*(27) = 10.756, *p* <.001).

*Developing readers.* Based on visual inspection of the scalp topography (Figure S3), we defined a sensor-level ROI which spanned across the following 10 parieto-occipital sensors: 055, 133, 135, 136, 138, 139, 140, 155, 156, 157.

At 6 Hz, results revealed a significant response in all conditions (see plot in Figure S4):

Condition 0 (*t*(16) = 9.567, *p* < .001);

Condition 1 (*t*(16) = 11.334, *p* <.001);

Condition 3 (*t*(16) = 9.467, *p* <.001).

Overall, these results represent a sanity check for the adopted paradigm and provide evidence for neural entrainment at the base stimulation frequency (6 Hz) in both skilled and developing readers.


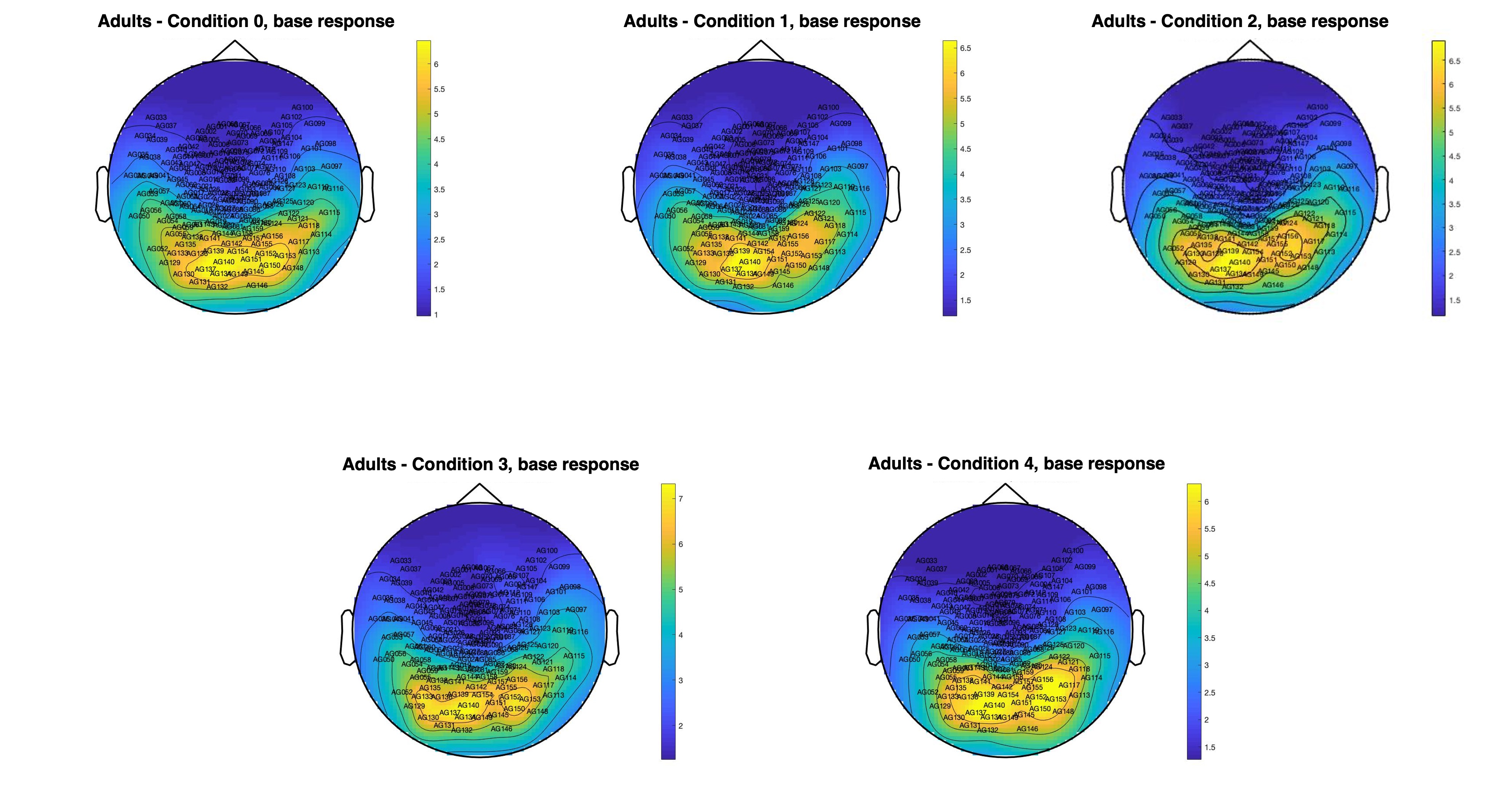


**Figure S1**. Topographic maps of sensor-level response to base frequency (6 Hz) in skilled adult readers, in all conditions.


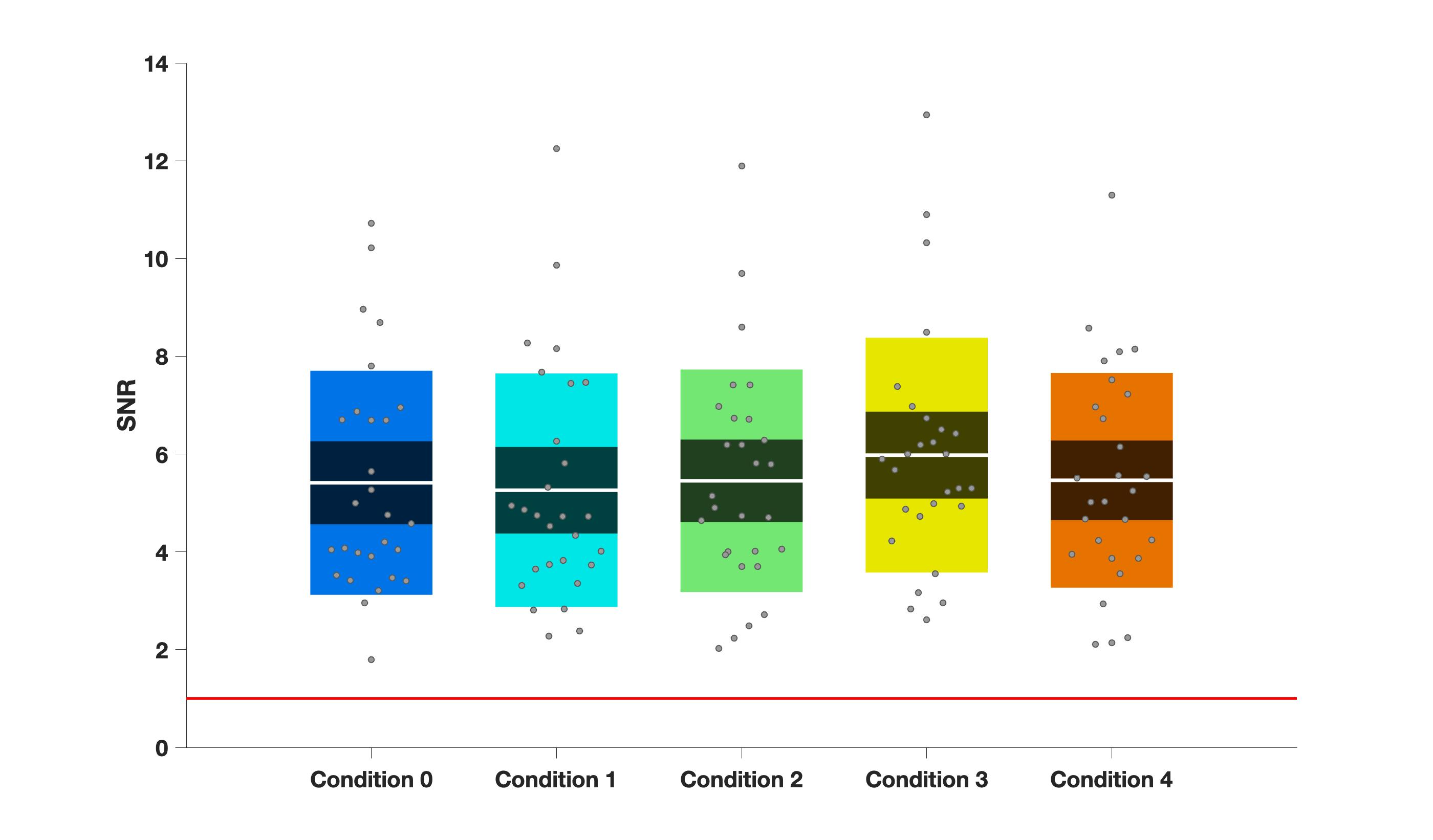


**Figure S2**. Visualization of the mean SNR of the base stimulation frequency (6 Hz) in skilled adult readers, by condition. Non-boxplots illustrating mean (white line), standard error of the mean (darker colored area), and standard deviation (lighter colored area) per condition (C0–C4). Points represent individual participants (N=28). The red line represents the noise level (1), against which SNR is compared in each condition. The grey dots illustrate individual participants’ average SNR of the oddball response in each condition.

The structure of the contrast between oddball (italic, bold) and base stimuli (italic) in each condition is as follows: Condition 0, ***words*** in *nonwords* (e.g., ***roll*** in *kltq*); Condition 1, ***stem+suffix*** in *nonstem+suffix* (***softity*** in *terpity*); Condition 2, ***stem+nonsuffix*** in *nonstem+nonsuffix* (***softert*** in *terpert*); Condition 3, ***stem+suffix*** in *stem+nonsuffix* (***softity*** in *terpert*); Condition 4, ***nonstem+suffix*** in *nonstem + nonsuffix* (***terpity*** in *terpert*).


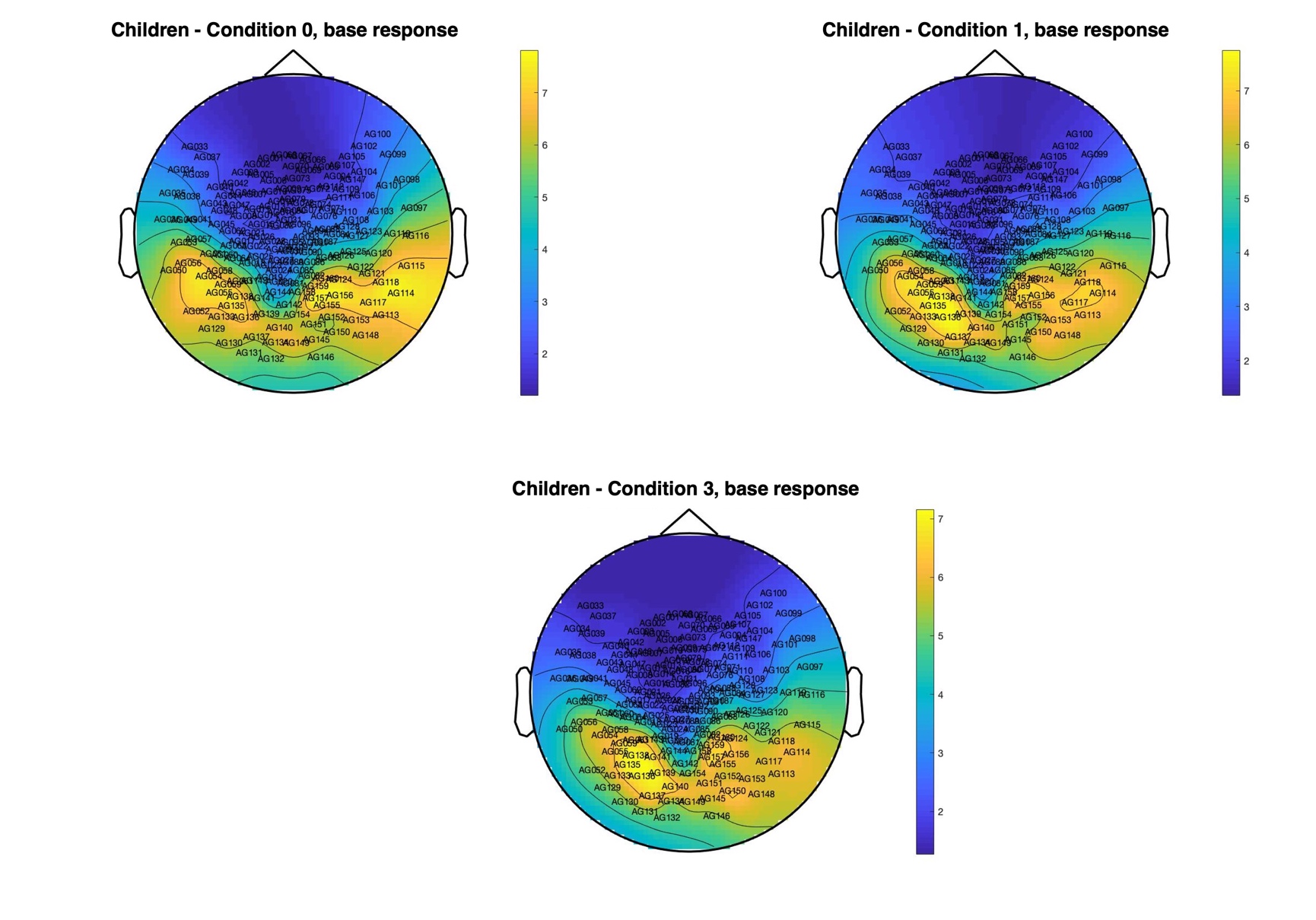


**Figure S3**. Topographic maps of sensor-level response to base frequency (6 Hz) in developing readers, in all conditions.


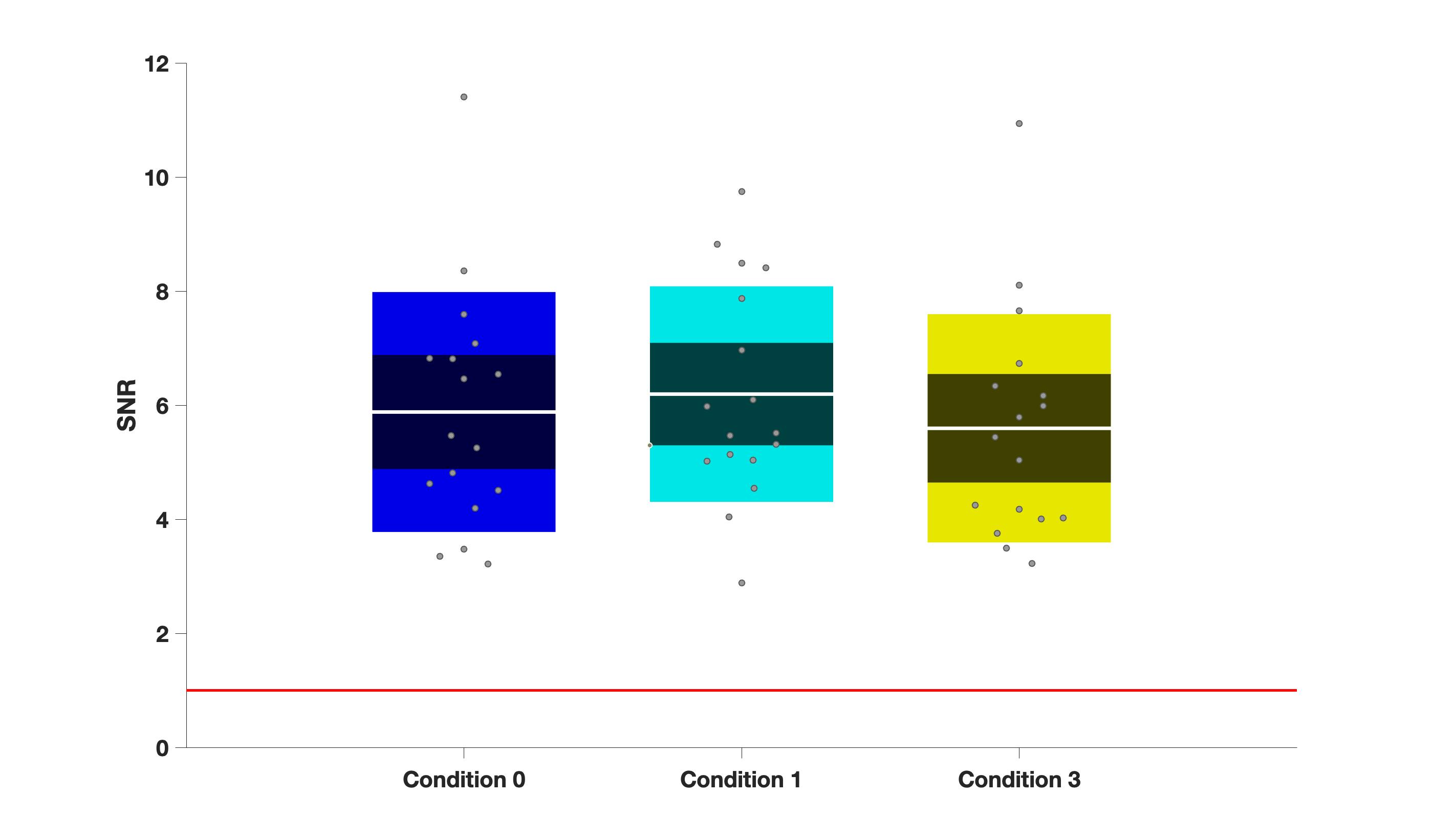


**Figure S4**. Visualization of the mean SNR of the base stimulation frequency (6 Hz) in developing readers, by condition. Non-boxplots illustrating mean (white line), standard error of the mean (darker colored area), and standard deviation (lighter colored area) per condition (C0, C1, C3). Points represent individual participants (N=17). The red line represents the noise level (1), against which SNR is compared in each condition. The grey dots illustrate individual participants’ average SNR of the oddball response in each condition.

The structure of the contrast between oddball (italic, bold) and base stimuli (italic) in each condition is as follows: Condition 0, ***words*** in *nonwords* (e.g., ***roll*** in *kltq*); Condition 1, ***stem+suffix*** in *nonstem+suffix* (***softity*** in *terpity*); Condition 3, ***stem+suffix*** in *stem+nonsuffix* (***softity*** in *terpert*).
